# Fungal–Mineral Interaction: Astrobiology Insights from Iron-Rich Mineral Alteration by an Extremophile Black Fungus

**DOI:** 10.1021/jacsau.5c01365

**Published:** 2025-12-15

**Authors:** Alef dos Santos, Fluvio Molodon, Júnia Schultz, Mauricio Augusto P. M. da Silva Alves, Alexandre Soares Rosado, Kurt Konhauser, Edson Rodrigues-Filho, Merve Yeşilbaş

**Affiliations:** † Department of Chemistry, 201361 Federal University of São Carlos, São Carlos 13565-905, Brazil; ‡ Department of Earth & Atmospheric Sciences, University of Alberta, Edmonton T6G 2E3, Canada; § Department of Chemistry, Umeå University, Umeå SE-90187, Sweden; ∥ Department of Ecology, Environment and Geoscience, Umeå University, Umeå SE-90187, Sweden; ⊥ Oceanographic Institute, University of São Paulo, São Paulo 05508-020, Brazil; # Biological and Environmental Science and Engineering Division, King Abdullah University of Science and Technology, Thuwal 23955, Saudi Arabia

**Keywords:** hematite, siderophores, mass spectrometry, metabolomics, biosignatures, redox processes

## Abstract

Iron-rich minerals, such as hematite (α-Fe_2_O_3_), are prominent constituents of the Martian surface; they are considered to be potential indicators of past aqueous activity and habitability. This study investigated the interaction between the extremophilic black fungus *Rhinocladiella similis* LaBioMMi 1217 and hematite under simulated laboratory conditions on Mars, focusing on redox-mediated dissolution processes, metabolic adaptations, and biosignature formation. The fungus was cultivated with powdered and polished hematite substrates, and mineral alteration was monitored through physicochemical measurements and scanning electron microscopy (SEM). Genome mining was performed to identify and map genes involved in iron metabolism. The metabolic profile of the fungus under hematite treatment was assessed via untargeted metabolomics. Over 15 days, the cultures exhibited marked acidification (pH decreased from 7.0 to 4.7) and a 10-fold increase in the dissolved Fe^2+^ ion concentration (26–270 mg/L), indicating metabolically driven iron reduction. SEM revealed surface etching and localized roughening consistent with microbially induced weathering, whereas these changes were absent in the abiotic controls. Genes linked to siderophore biosynthesis (*sidA*, *sidC*, *sidD*, *sidF*, *sidH*, *sidI*, and *sidL*) and reductive iron assimilation (*FET3*, *FTR1*, and *FRE1*) were identified. Untargeted metabolomics confirmed the secretion of organic acids, iron-chelating siderophores (e.g., ferrichrome C), and redox-active aromatic compounds in the presence of hematite, supporting a multifaceted strategy that combines acidification, chelation, and redox mediation. Collectively, these results show that the fungus actively promotes hematite dissolution through organic molecule-mediated mechanisms. Such interactions hold astrobiological relevance, as fungal modification of hematite might lead to the production of diagnostic chemical and mineralogical biosignatures, informing future life-detection strategies on Mars.

## Graphical abstract

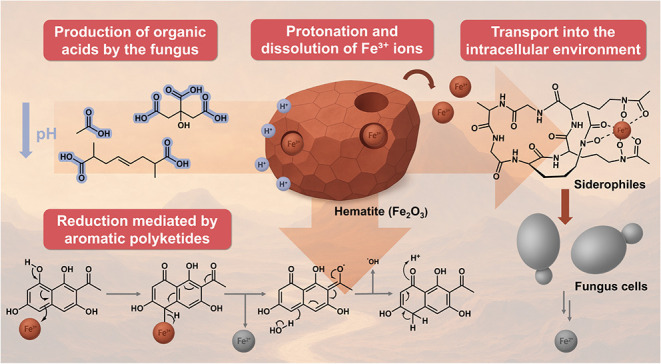

## Introduction

The presence of hematite (α-Fe_2_O_3_) on the Martian surface and subsurface has been widely documented by orbital and surface missions, including *Mars Global Surveyor*, *Mars Exploration Rovers*, and *Perseverance*;^1–4^ this mineral has been detected in extensive deposits, such as those in Meridiani Planum, Aram Chaos, and Valles Marineris.^5–7^ Hematite formation is associated with multiple pathways, including direct precipitation from iron (Fe)-rich solutions, hydrothermal transformation of ferric precursors, and thermal dehydroxylation of iron oxyhydroxides.^5,8^ These routes often reflect previous aqueous and potentially habitable conditions, giving hematite and other iron oxide minerals central roles in paleoenvironmental reconstruction and Martian astrobiology. Its chemical stability and distinct spectral properties further support its relevance as both a recorder of ancient environmental conditions and a substrate for potential microbial redox processes.^6,9–11^

On Earth, hematite and related iron oxides are closely associated with microbial activity in environments that serve as analogs for past Martian settings. In sites such as the Río Tinto Basin (Spain), which is characterized by extreme acidity, high dissolved iron concentrations, and active iron cycling—conditions analogous to those inferred for ancient Mars—molecular biomarkers and microfossil-like structures are preserved within iron oxide matrices.^9,10,12^ Similar preservation has been reported in other Fe-rich systems, such as banded iron formations, hydrothermal deposits, lateritic soils, and rock varnish, where hematite can entomb microbial cells and extracellular polymeric substances at micrometer scales.^13,14^ Such analogs highlight the dual relevance of hematite on Mars as a mineralogical indicator of past water–rock interactions and a potential long-term repository of biosignatures accessible to future sample return missions.

Microbe–mineral studies have demonstrated that bacteria and archaea can oxidize Fe­(II) or reduce Fe­(III), altering their physical and chemical properties through mechanisms involving direct electron transfer, chelation, or extracellular electron shuttles.^15–18^ These redox transformations can alter mineral crystallinity, morphology, and elemental composition, sometimes resulting in the precipitation of secondary minerals that entomb microbial cells or their organic exudates.^19^ Such processes both drive biogeochemical cycles and leave mineralogical and chemical fingerprints that can persist over geological timescales, thereby providing potential biosignatures detectable in the rock record.^20^ Extrapolated to Mars, these mechanisms suggest that microbe–hematite interactions could have occurred wherever liquid water and Fe-bearing minerals coexisted, given hematite’s wide distribution across diverse Martian geochemical settings. Combined with its stability and capacity to preserve morphological and chemical traces of biological activity, hematite remains a compelling target for life-detection missions.^21,22^

Despite the significant focus on prokaryotic systems, the role of eukaryotic microorganisms, particularly fungi, in modifying iron-bearing minerals remains comparatively underexplored. Fungi are among the most versatile and stress-tolerant organisms, thriving in a wide range of extreme habitats that mimic extraterrestrial conditions, including hyperarid deserts, hypersaline waters, acidic mine drainages, and Antarctic permafrost.^23^ Their ability to withstand multiple concurrent stresses (e.g., low water activity, high salinity, extreme temperatures, and intense radiation) often surpasses that of several prokaryotes, owing to adaptations such as melanized cell walls, osmolyte accumulation, and meristematic growth, as well as the capacity to enter dormant but rapidly reversible states. These traits make fungal metabolism compelling candidates in astrobiology for evaluating the potential limits of eukaryotic life beyond Earth.^24^ Furthermore, existing evidence shows that fungi can mobilize metals, produce chelating molecules, and mediate redox reactions that influence iron mineral stability and transformation.^25–27^ These capabilities suggest that fungus–mineral interactions could be highly relevant in extraterrestrial contexts, particularly in Martian environments with abundant iron mineral content and intermittent water activity.^28,29^

With their high prevalence in extreme environments on Earth, metabolic versatility, and increasingly recognized role in astrobiology, fungi are valuable models for studying microbe–mineral interactions under planetary analog conditions. Thus, this study investigated the bidirectional relationship between *Rhinocladiella similis* LaBioMMi 1217, a eukaryotic astrobiological model,^30,31^ and hematite, specifically examining the ability of the fungus to modify hematite’s physical, chemical, and structural properties and the subsequent influence of hematite on the fungal metabolic profile as revealed through integrated multiomics approaches. Collectively, these results provide a comprehensive assessment of how such interactions can affect the formation, preservation, and detectability of biosignatures, providing direct implications for life-detection strategies in current and future Mars exploration missions.

## Materials and Methods

### Microbial Characterization and Genome Mining

The polyextremophile strain *R. similis* LaBioMMi 1217, which was previously isolated and characterized,^30,32^ was used in this study. This strain was selected because of its remarkable ability to thrive in iron-rich mineral environments, as demonstrated in previous studies. The genome of *R. similis* LaBioMMi 1217 was previously sequenced, assembled, and characterized by dos Santos et al., and a growth curve was established prior to the experimental assays (Figure S1). In this study, we extended their efforts through genome mining analyses to explore the genetic potential of the strain for astrobiologically relevant metabolism and metabolite biosynthesis. Hence, the assembled FASTA file of the genome of *R. similis* LaBioMMi 1217 was used for genome mining to track a set of genes related to iron metabolism.

The gene set was defined according to literature searches across well-described iron pathways. Once defined, the gene-translated protein sequences of interest were systematically retrieved from UniProtKB by using a custom Python script. Sequences, sets, and scripts are available at https://github.com/modolon/Genome_mining_R_similis. Briefly, the script queried the UniProtKB REST API using protein names and organism-specific taxonomic IDs provided in an input file, applying relaxed filters (annotation score ≥1) to maximize matches. For each successful query, metadata (e.g., accession ID, gene names, and sequence length) were compiled into a structured CSV file, whereas the corresponding FASTA sequences were downloaded and saved as individual text files in a dedicated directory. Unmatched queries were logged for further review.

Once retrieved, the sequences were manually checked, dereplicated, and implemented in an annotation pipeline. First, a BLAST database was generated from the assembled genome using makeblastdb, and all protein queries were searched against it with tBLASTn (e-value cutoff: 1 × 10^–5^). Output files were parsed to extract significant hits, retaining only alignments with e-values <1 × 10^–5^, which were compiled into a summary file of putative unannotated regions. Significant hits from both approaches were compiled and cross-referenced with genomic coordinates from the GBK file. Finally, putative homologous genes with ≥40% identity were visualized using the gggenes package (v0.5.1) in R (The R Foundation for Statistical Computing, Vienna, Austria) to map their genomic position and strand orientation.

### Fabrication and Characterization of Polished Hematite Chips

Hematite chips were prepared from raw mineral collected in Alberta, Canada. The raw material was cut into strips measuring 1 × 5 cm with a thickness of 0.2 cm. These strips were polished by using a mechanical polishing machine, starting with coarse-grit abrasives and progressively moving to finer grits until a smooth, highly polished surface was obtained.

For mineralogical characterization by X-ray diffraction (XRD), approximately 10 g of raw hematite was processed in a ball mill. The milling conditions were adjusted to ensure efficient particle size reduction, and samples were ground for 5 min to obtain a fine, homogeneous powder.

### Cultivation and Microscopy of Black Yeast on a Substrate Enriched with Hematite

To investigate changes in the fungal metabolic profile, *R. similis* LaBioMMi 1217 was cultivated in a hematite-enriched medium using 50 mL Erlenmeyer flasks, each containing 20 mL of potato dextrose broth (PDB; Sigma-Aldrich, St. Louis, MO, USA) and 500 mg of hematite powder. The inoculation was performed by adding 100 μL of a cell suspension previously prepared at 1 × 10^4^ cells/mL. As a control, a separate set of flasks containing only the fungus in 20 mL of PDB was prepared. To account for background signals from the culture medium and hematite during data analysis, blank experimental groups with no fungal inoculum were included. All flasks were incubated at 25 °C under orbital agitation (150 rpm) for 15 days. The experiments were conducted in five replicates (*n* = 5). The same experimental setup was used to monitor ferrous ion (Fe^2+^) concentrations and pH variation over time, albeit with three replicates (*n* = 3).

To verify the morphological changes in the hematite mineral, three polished hematite chips measuring approximately 1 × 5 × 0.2 cm were placed in PDB medium. The aforementioned inoculum was employed, and the fungus was grown under the same conditions as those used for the metabolomic analysis. As an abiotic control, three hematite chips were placed under the same conditions without adding the fungal inoculum.

After fungal growth was permitted, the chips were removed, air-dried, and fixed in 3% glutaraldehyde for 3 h. The fixed samples were dehydrated using a series of isopropyl alcohol gradients (35%, 50%, 75%, 90%, and 100%), followed by critical point drying for 5 h and coating with amorphous carbon particles. Images were acquired by scanning electron microscopy (SEM; Zeiss Sigma 300 VP-FESEM, Zeiss, Oberkochen, Germany), located at the University of Alberta

### Fe­(II) Concentration Measurements and pH Analysis

The Fe^2+^ concentration in the culture medium was determined using spectrophotometry based on the complexation method with 1,10-phenanthroline. Analyses were conducted over 15 days, with sample collection performed every 3 days. For each measurement point, 1 mL of the sample was collected under sterile conditions and immediately centrifuged at 5000 rpm to clarify the samples and remove cells and particulate matter, thereby minimizing potential interference in the subsequent 1,10-phenanthroline reaction. After centrifugation, 500 μL of the supernatant were transferred to a 50 mL volumetric flask, and the volume was adjusted with acetate buffer. Subsequently, 1 mL of the diluted solution was withdrawn and mixed with 1 mL of 0.1% (w/v) 1,10-phenanthroline. The samples were incubated at room temperature for 10 min before absorbance measurements using a Nanocolor 500D spectrophotometer (MACHEREY-NAGEL, Düren, Germany), with 1 cm path length cuvettes and detection at 510 nm. A blank containing only acetate buffer and the 1,10-phenanthroline reagent was prepared and measured under the same conditions to correct for the background absorbance. Fe^2+^ was quantified by interpolation on a calibration curve constructed from FeSO_4_ standard solutions prepared at concentrations of 0.5, 1, 2, 4, and 5 mg/L. The curve was obtained by fitting the experimental values to a linear regression, ensuring an appropriate coefficient of determination (*R* ^2^) for quantitative analysis (Figure S2). The obtained results were subsequently multiplied by the dilution factor to determine the actual iron concentration in the original sample. Additionally, the pH was measured using the remaining sample volume with a pH meter (Mettler Toledo, Greifensee, Switzerland).

### XRD Analyses

Powder XRD patterns were collected using a Cu Kα radiation source (λ = 1.5406 Å) with a 2θ angular resolution of 0.02° and a LynxEye XE-T 1D detector (Bruker, Billerica, MA, USA), located at Umeå University. Pure hematite, abiotic control, and hematite + fungus samples were finely ground and mounted on a zero-background Si holder to minimize preferred orientation effects. The diffraction data were processed in OriginPro 2024 (https://www.originlab.com/index.aspx?go=Products/Origin), including background subtraction, peak smoothing (Savitzky–Golay algorithm), and peak fitting with pseudo-Voigt functions. Phase identification was conducted by comparing experimental peaks with reference patterns from the ICDD PDF-4+ database. Lattice parameters and crystallite sizes were estimated from the refined diffraction profiles.

### Extraction of Microbial Metabolites

To assess the production of secondary metabolites induced by the interaction between *R. similis* LaBioMMi 1217 and hematite, mycelial biomass and culture supernatant were collected from fungal cultures and controls in 100 mL Erlenmeyer flasks into 50 mL polypropylene centrifuge tubes. Samples were immediately frozen at −80 °C for 24 h to preserve labile compounds and subsequently lyophilized to complete dryness. Metabolite extraction was performed via solvent-assisted cell lysis with 10 mL of a methanol:water solution (80:20, v/v) added to each tube. Samples were further subjected to mechanical agitation on a vertical orbital shaker for 30 min, followed by sonication in an ice-cooled ultrasonic bath for 15 min to promote efficient cell disruption and metabolite release.

To ensure consistency across treatments and avoid bias in subsequent statistical analyses, an equal amount of hematite was added to the control group during extraction. Following extraction, 1 mL of each supernatant was transferred to a 1.5 mL microcentrifuge tube and evaporated to dryness under reduced pressure by using a vacuum concentrator. The dried residues were reconstituted in 250 μL of a methanol:water solution (1:1, v/v), vortexed, and centrifuged before analysis by ultrahigh-performance liquid chromatography coupled to high-resolution tandem mass spectrometry (UHPLC–HRMS/MS). A detailed schematic of the workflow is provided in Figure S3.

### UHPLC–HRMS/MS Analysis

An untargeted metabolomic study of the hydroalcoholic crude extracts was performed by using a 1290 Infinity UHPLC system (Agilent Technologies, Santa Clara, CA, USA), located at Umeå University. Two microliters of each sample were injected onto an Acquity UPLC HSS T3, 2.1 mm × 50 mm, 1.8 μm C18 column (Waters, Milford, MA, USA) in combination with a 2.1 mm × 5 mm, 1.8 μm VanGuard precolumn (Waters Corporation), held at 40 °C. The gradient elution buffers were A (0.1% formic acid in H_2_O) and B (0.1% formic acid in 75/25 acetonitrile:2-propanol), and the flow rate was 0.5 mL/min. The compounds were eluted with a linear gradient consisting of 0.1%–10% B over 2 min, B increasing to 99% over 5 min and held at 99% for 2 min, and B decreasing to 0.1% for 0.3 min. Then, the flow rate was increased to 0.8 mL/min for 0.5 min, held for 0.9 min, and then reduced to 0.5 mL/min for 0.1 min before the next injection. The compounds were detected using a 6550 Q-TOF mass spectrometer (Agilent) equipped with a jet stream electrospray ion source operating in positive or negative ion mode. The settings were identically maintained across both modes, excluding the capillary voltage. A reference interface was connected to the system for accurate mass measurements. The nozzle voltage was 300 V, the fragmentor voltage was 380 V, the skimmer was 45 V, and the OCT 1 RF Vpp was 750 V. The *m*/*z* range was 70–1700, and data were collected in centroid mode with an acquisition rate of 4 scans/s (1977 transients/spectrum). MS/MS analyses were performed using a collision energy ramp ranging from 15 to 45 eV. Data-dependent auto MS/MS acquisition targeted the six most intense precursor ions. A pooled sample generated from all injections was included as a quality control (QC) and injected at regular intervals to monitor analytical stability. A blank solvent injection was run at the beginning of each batch to rule out carryover, and no peaks indicative of cross-contamination were observed. All injections were randomized to avoid analytical bias. Each experimental group was analyzed with 5 biological replicates, including an equivalent number of blanks and controls.

### MS Data Processing

The UHPLC–HRMS/MS data were processed using MZmine 3,^33^ following established workflows for untargeted metabolomics. Raw data files were first converted to the .mzML format with MSConvert (ProteoWizard, https://proteowizard.sourceforge.io/) and subsequently imported into MZmine (https://mzio.io/mzmine-news/) for feature detection, alignment, and annotation. A complete description of the processing parameters (e.g., thresholds, noise level, filters) is provided in Table S1.

The resulting aligned feature list was exported as a .csv file and uploaded to MetaboAnalyst 5.0 ^34^ for statistical analysis. Exploratory data analysis was performed via principal component analysis (PCA) to evaluate analytical reproducibility and data set structure. Univariate analysis included analysis of variance with a Benjamini–Hochberg false discovery rate correction for multiple testing. Volcano plots were generated to highlight significantly altered features between the experimental groups.

Metabolite annotation was achieved through spectral library matching against public databases using the Global Natural Products Social Molecular Networking platform (GNPS; https://gnps2.org) and molecular networking approaches.^35^ SIRIUS was employed for in silico fragmentation, molecular formula prediction, and compound class annotation via CANOPUS.^36^ Manual curation was conducted when required to refine assignments and resolve ambiguous identifications, ensuring high-confidence metabolite annotation.

## Results

### pH Variation and Fe^2+^ Mobilization during Fungus–Hematite Interactions

To investigate the potential of *R. similis* LaBioMMi 1217 to alter the structural and chemical properties of hematite, temporal changes in pH and dissolved Fe^2+^ concentrations were monitored under both biotic and abiotic conditions. Three experimental setups were employed: fungus incubated with hematite powder, an abiotic control containing only hematite in culture medium, and a biological control containing only the fungus in culture medium.

In the fungus–hematite system, pH exhibited a pronounced decrease over time, falling from 7.01 to 4.70 over 15 days of incubation (Figure 1A). Although an acidification trend was also observed in the biological control containing only the fungus, the magnitude of the decrease was less pronounced, indicating that the presence of hematite enhanced the process. This potentiating effect indicates that the fungus–mineral interaction accompanies and amplifies the acidification driven by fungal metabolism. Conversely, the pH remained stable in the abiotic control, with minor fluctuations and no sustained decrease, confirming that hematite, in the absence of biological activity, does not significantly contribute to medium acidification.

**Figure 1:**
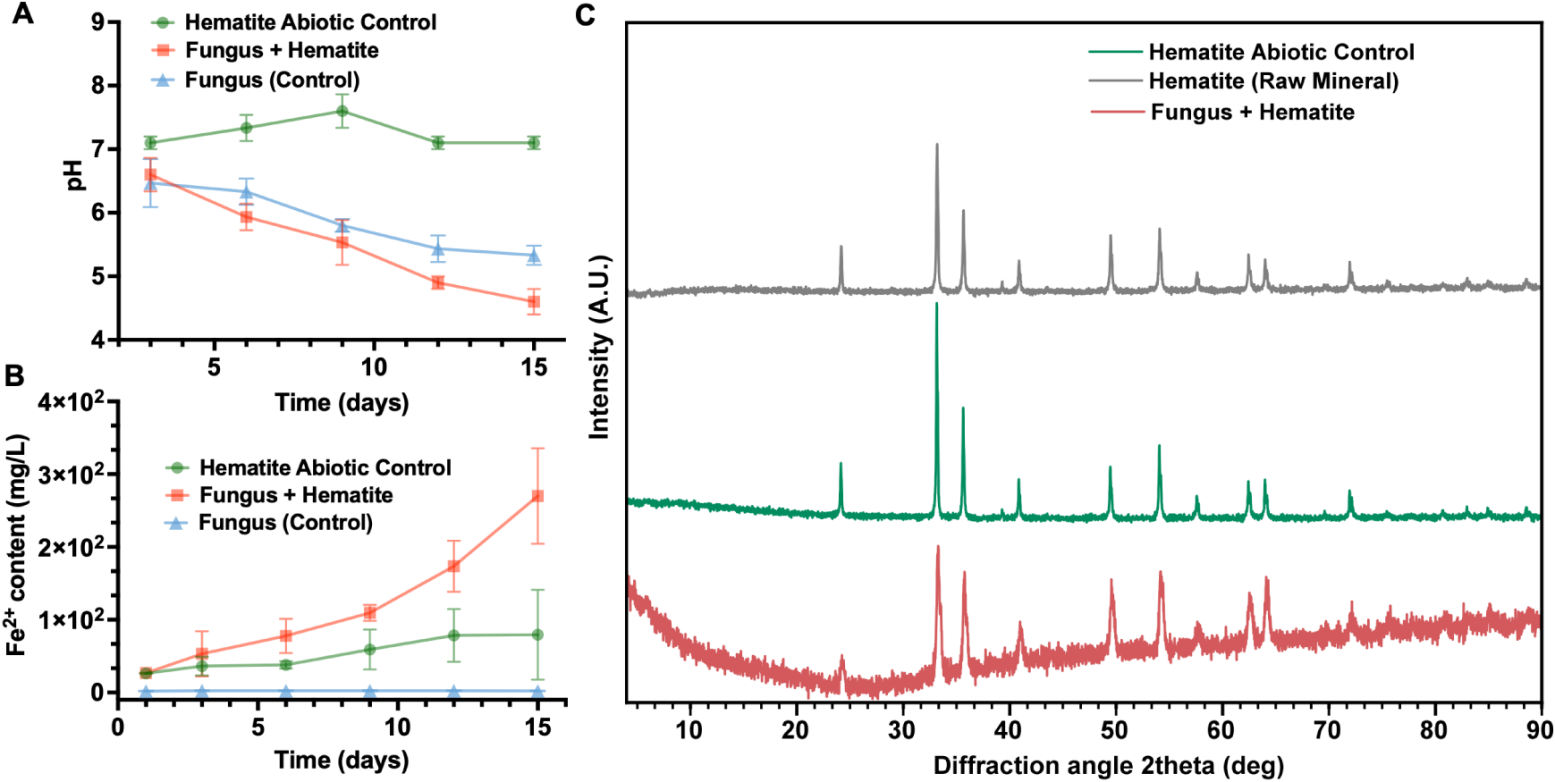
Temporal variations in the pH, Fe^2+^ ion mobilization, and crystalline structure of hematite under biotic and abiotic conditions. (A) pH variation and (B) Fe^2+^ mobilization over 15 days under different experimental conditions (*n* = 3). Three systems were analyzed: (●) hematite abiotic control, hematite incubated in PDB medium without fungal inoculation; (▲) fungus (control), *R. similis* LaBioMMi 1217 cultivated in PDB medium without hematite; (■) fungus + hematite (main experiment), *R. similis* LaBioMMi 1217 cultivated in the presence of hematite powder. Error bars represent standard deviations from biological replicates. (C) XRD patterns of raw hematite (black), hematite after abiotic incubation (green), and hematite after incubation with the fungus (red).

The changes in the dissolved Fe^2+^ concentrations clearly distinguished the groups (Figure 1B). In the fungus–hematite system, Fe^2+^ content increased markedly over time, from 26 mg/L on day 1 to 109 mg/L on day 9 and 270 mg/L on day 15. This substantial release suggests a biologically mediated dissolution of hematite, likely driven by fungal metabolic activity and associated acidification. By contrast, the abiotic control released only minimal Fe^2+^, and the biological control maintained baseline levels throughout the experiment, indicating negligible iron mobilization in the absence of fungus–mineral interactions.

XRD analyses further supported these findings (Figure 1C). The raw hematite sample displayed sharp, well-defined peaks characteristic of highly crystalline hematite, consistent with the reference pattern JCPDS 33-0664. The abiotic control preserved the principal diffraction features of hematite, with only subtle changes in peak intensity and width, indicating minimal surface or structural alterations. In stark contrast, hematite exposed to the fungus exhibited pronounced peak attenuation and an elevated amorphous background, particularly between 10° and 30° 2θ, indicative of partial loss of crystallinity. These changes are consistent with bioweathering, reflecting structural disruption and mineral dissolution induced by fungal metabolism.

### Mineral Morphological Characterization by SEM

To assess whether *R. similis* LaBioMMi 1217 induces structural modifications in hematite, polished mineral surfaces were examined after 15 days of incubation using SEM. In the abiotic control (Figure 2A,B), the surface appeared largely intact, displaying only minor scratches attributable to the polishing process and showing no evidence of significant morphological alterations.

**Figure 2:**
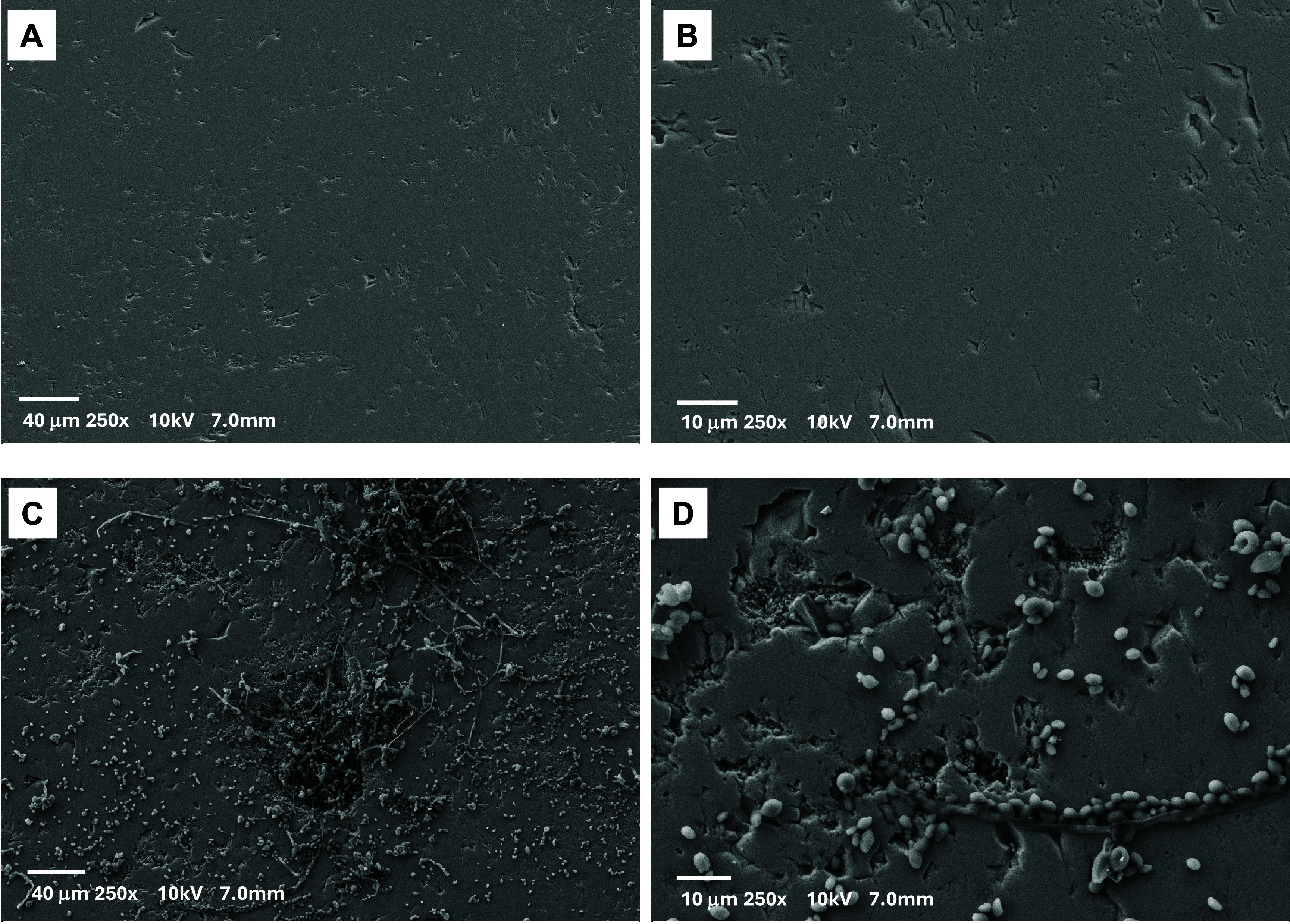
SEM images of hematite surfaces after 15 days of incubation. (A, B) Abiotic control: hematite incubated in PDB medium without fungal inoculation, revealing a smooth surface with minor polishing marks. (C, D) Fungal treatment: hematite incubated with *R. similis* LaBioMMi 1217, featuring abundant yeast-like cells, filamentous structures, and localized surface alterations, including increased roughness and depressions. All images were captured at 250× magnification under an accelerating voltage of 10 kV.

Conversely, hematite incubated with the fungus (Figure 2C,D) exhibited marked changes in the surface morphology. Numerous yeast-like cells were adhered to the mineral, often accompanied by filamentous extensions suggestive of hyphal structures or extracellular matrix components. The surface also displayed localized regions of increased roughness, depressions, and pitting—features absent in the abiotic control. These modifications were consistent with bioweathering, indicating that fungal activity might promote localized mineral dissolution and leaching of surface material into the culture medium.

### Iron Metabolism and Siderophore Biosynthesis Genes In *R. similis* LaBioMMi 1217

Genome mining identified 13 genes associated with iron metabolism in *R. similis* LaBioMMi 1217, all with strong statistical support (*p* < 0.05; Supplementary Table S2). These genes encode proteins involved in iron acquisition, transport, and homeostasis, including transporters (ARN1 and STR1), oxidases (*FET3* and *FETC*), permeases (FTR1 and FTRA), reductases (FRE1), and transcriptional regulators (SEF1 and SFU1). Within this set, we detected siderophore-related transporters (ARN1 and STR1) and the reductive iron assimilation system (*FET3/FTR1* and *FETC/FTRA*), indicating the genomic potential for multiple high-affinity uptake strategies. The full annotation is provided in Supplementary Data 1.

Given the presence of siderophore-associated transporters, we analyzed genes involved in siderophore biosynthesis, revealing the complete set of genes for Sid family members, including *sidA*, *sidD*, *sidF*, *sidG*, *sidH*, *sidI*, and *sidL*, with 40 high-confidence hits (e-value <1 × 10^–5^) distributed across the genome. Most gene copies with high homology (percent identity ≥ 40%) were concentrated in two contigs: JAZDCV010000006.1 (11 genes; 36.7% of the total) and JAZDCV010000005.1 (five genes; 16.7%). The arrangement differed markedly between loci. In JAZDCV010000005.1, *sidA* was located at 805,352–806,749 bp on the forward strand (+), separated by an extensive intergenic region of approximately 740 kb from a downstream reverse-strand (−) cluster containing *sidD*, *sidF* (two copies), *sidL*, and *sidH* (1,547,857–1,554,417 bp). Conversely, JAZDCV010000006.1 presented a compact region of approximately 13 kb (1,331,972–1,345,133 bp) with six tandem *sidI* copies interspersed with *sidD* and *sidF*, all on the reverse strand (−) and separated by short intergenic spacers (30–50 bp), suggesting local duplication events.

In total, 54 *sidC*-like sequences were annotated in the *R. similis* LaBioMMi 1217 genome, all of which displayed <40% identity to the reference protein, indicating substantial sequence divergence. Given that the target siderophore is indeed produced by this strain, it is likely that at least one of these divergent copies retains *sidC*’s functional role.

The organization and orientation of siderophore biosynthesis genes in the two primary contigs of the fungi are illustrated in Figure 3, and complete genomic coordinates are provided in Supplementary Data 1.

**Figure 3:**
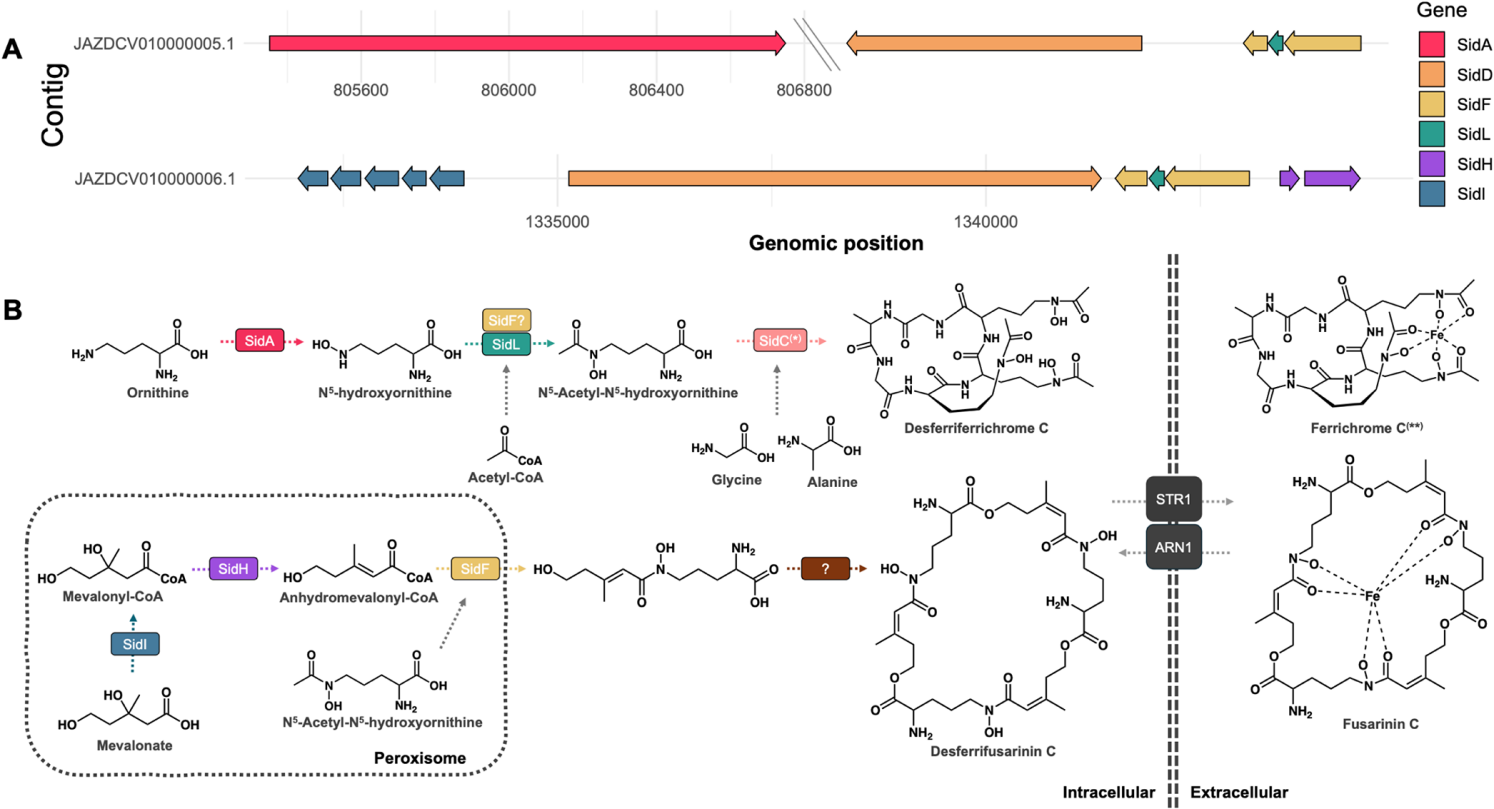
Gene organization and predicted pathway for siderophore biosynthesis and transport in *R. similis* LaBioMMi 1217. (A) Schematic representation of the organization of siderophore biosynthesis genes in the *R. similis* LaBioMMi 1217 genome. In contig JAZDCV010000005.1, the *sidA* gene (805,352–806,749 bp) is located in isolation at one end, followed by a large intergenic region (approximately 740 kb, indicated by the axis break) preceding a gene cluster containing *sidD*, *sidF*, *sidL*, and *sidH* (1,547,857–1,554,417 bp). The arrow direction indicates gene orientation: *sidA* on the forward strand (+) and the others on the reverse strand (−). (B) Proposed biosynthetic pathway inferred from genome mining data for iron metabolism in *R. similis* LaBioMMi 1217, culminating in the production of siderophores. The colored rectangles indicate the proteins encoded by *sidA*, *sidC*, *sidD*, *sidF*, *sidH*, *sidI*, and *sidL* genes. (*) The gene encoding *sidC* was detected with a percent identity below the 40% cutoff. (**) Ferrichrome C detected by LC–MS/MS.

### Untargeted Metabolomics

To evaluate the impact of hematite on the metabolic profile of *R. similis* LaBioMMi 1217, untargeted UHPLC–HRMS/MS analyses were performed in negative and positive ion modes. Crude extracts from cultures grown in the presence of powdered hematite (fungus + hematite) and control cultures grown only in PDB medium (fungus) were compared. QC samples, prepared by pooling aliquots from all extracts, were included to monitor analytical reproducibility.

In negative ion mode, PCA revealed that the first two principal components (PC1 and PC2) accounted for 74.3% of the total variance (47.2% for PC1 and 27.1% for PC2), revealing a clear separation between the two experimental groups (Figure 4A). Control samples (fungus) clustered in the lower-left quadrant, whereas samples from the fungus + hematite condition grouped in the upper-left quadrant. QC samples were tightly clustered near the origin, confirming consistent instrument performance. The corresponding volcano plot (Figure 4B) highlighted extensive metabolic differences among the conditions, with 103 significantly upregulated features and 94 significantly downregulated features in the presence of hematite (fold change ≥2, *p* < 0.05). Several discriminant ions were annotated with their respective *m*/*z* values and retention times (RTs), including *m*/*z* 173.0464 (1.51 min) and *m*/*z* 327.1274 (4.70 min), which were upregulated in the hematite-treated cultures.

**Figure 4:**
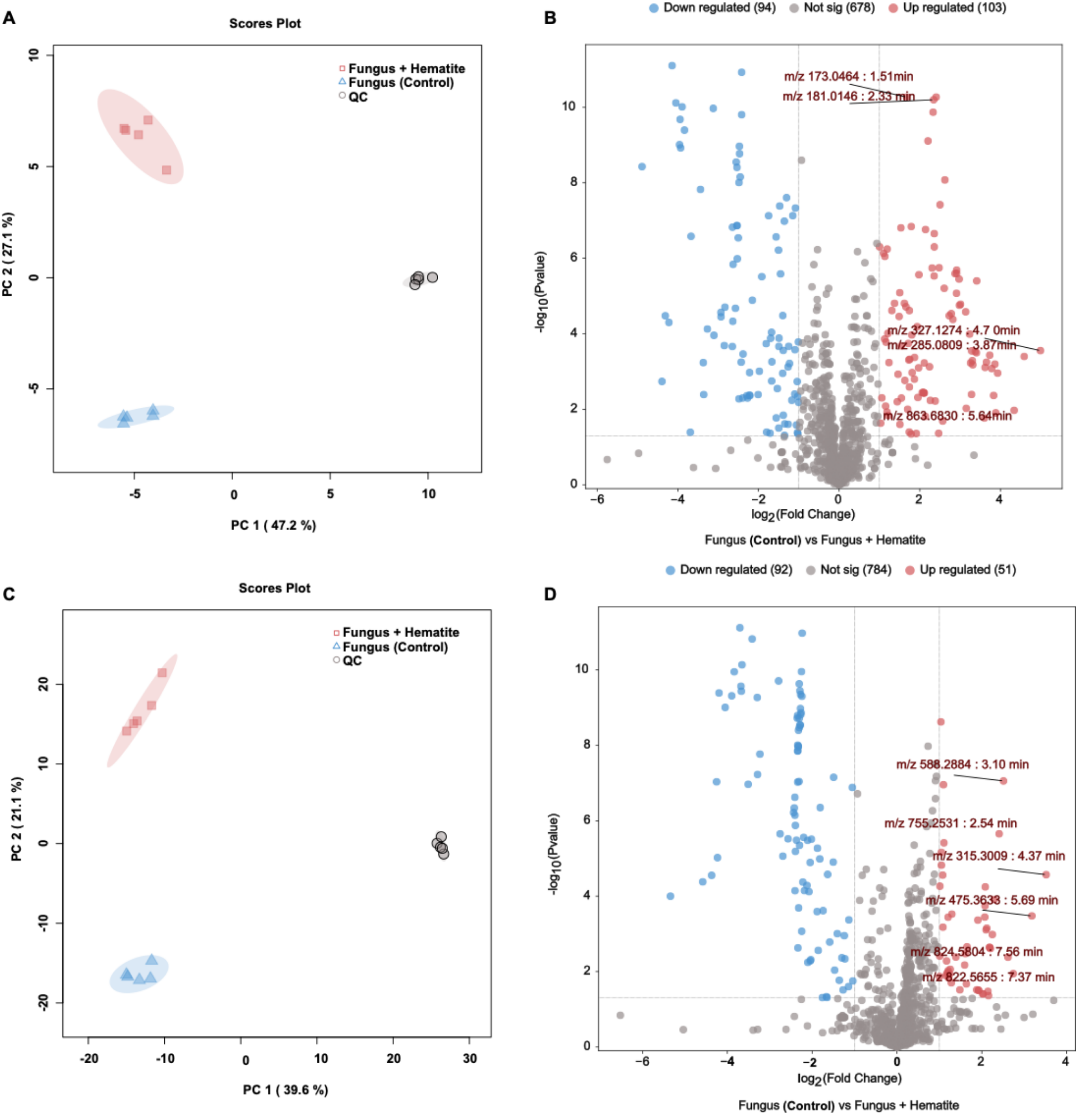
Untargeted metabolomics analysis of *R. similis* LaBioMMi 1217 cultivated with and without hematite as acquired by UHPLC–MS/MS in the negative (A–B) and positive (C–D) ion modes. (A, C) PCA score plots revealing distinct clustering among cultures grown in the presence of hematite (fungus + hematite, red), control cultures without hematite (fungus, blue), and pooled QC samples (QC, gray). Percentages in parentheses indicate the proportion of total variance explained by each component. (B, D) Volcano plots displaying the distribution of features according to log_2_ fold change (fungus + hematite vs fungus) and – log_10_(p-value). Red dots indicate significantly upregulated features (fold change ≥2, *p* < 0.05), blue dots indicate significantly downregulated features (fold change ≤ – 2, *p* < 0.05), and gray dots represent nonsignificant features. Selected discriminant *m*/*z* values are annotated with their retention times.

In the positive ion mode, PCA indicated that PC1 and PC2 explained 60.7% of the total variance (39.6% for PC1 and 21.1% for PC2), again revealing distinct clustering among the treatments (Figure 4C). Control samples grouped in the lower-left quadrant, whereas fungus + hematite samples occupied the upper-left quadrant. QC samples formed a compact cluster near the center of the plot, further demonstrating analytical reproducibility. The volcano plot for the positive ion mode (Figure 4D) identified 51 upregulated and 92 downregulated features under hematite exposure, with notable *m*/*z* values of 568.2884 (RT 3.10 min) and 475.3538 (RT 5.69 min). These results indicate that exposure to hematite induces marked alterations in the metabolome of *R. similis* LaBioMMi 1217, negatively and positively affecting ionizing compounds.

Untargeted metabolomics revealed a chemically diverse metabolome in *R. similis* LaBioMMi 1217, encompassing primary and specialized metabolites across positive and negative electrospray ionization (ESI) modes. Annotation based on GNPS spectral libraries, *in silico* fragmentation (SIRIUS), and manual curation from the literature enabled the identification of 47 major metabolites distributed among 14 chemical families (Table S3), including betaine lipids, glycerophospholipids, oxylipins, phenolic acids, siderophores, aromatic polyketides, carotenoid derivatives, and small peptides.

The lipidome was composed of glycerophospholipids, such as phosphatidylcholines [PC (16:0/18:1), PC (18:2/18:2)], phosphatidylethanolamines, lysophosphatidylcholines (LPC 18:1, LPC 18:2), and phosphatidic acids [PA (16:0/18:2), PA (18:2/18:2)]. Betaine lipids, including diacylglyceryltrimethylhomoserine derivatives (DGTS and DGTSA), were also detected.

Specialized metabolites included siderophores such as ferrichrome C, ferrichrocin, and their desferri analogs; aromatic polyketides such as 1-(1,3,6,8-tetrahydroxynaphthalen-2-yl)­ethan-1-one and related naphthoquinones; oxylipins (e.g., 12,13-DiHOME, 9­(S)-HOTrE); and carotenoid derivatives such as 2,6,10,15,19,23-hexamethyltetracosa-6,10,14,18-tetraene-2,3,22,23-tetraol.

The comparative molecular network feature maps (Figure 5) illustrate compositional differences between the control and hematite-exposed cultures. In the ESI­(+) mode, hematite exposure was associated with changes in siderophore-related ions and phospholipids, whereas the ESI(−) mode highlighted differences in carboxylic acids, oxylipins, and aromatic polyketides. The feature-based molecular networking (FBMN) approach was performed using the GNPS platform, which organizes MS/MS spectra into networks based on spectral similarity. Each node corresponds to a molecular feature, and edges connect features with cosine similarity scores above a defined threshold, thereby visualizing chemical relationships and putative structural analogs within the metabolome.

**Figure 5:**
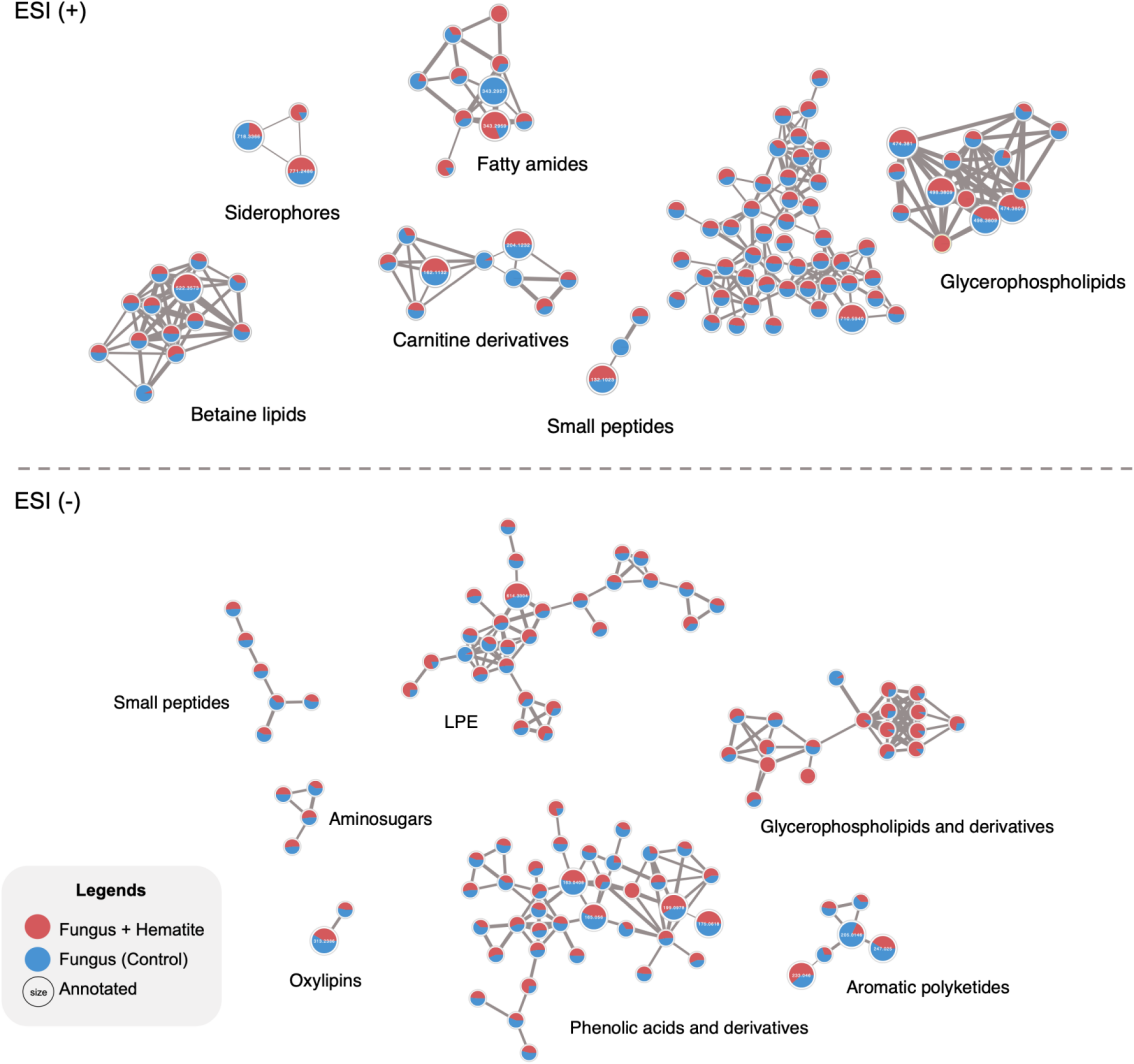
FBMN of *R. similis* LaBioMMi 1217 cultivated in the presence (red nodes) and absence (blue nodes) of hematite, generated from UHPLC–MS/MS data acquired in positive (ESI+, top) and negative (ESI–, bottom) ionization modes. Each node represents a distinct molecular feature, and edges indicate MS/MS spectral similarity (cosine score ≥0.7). Node coloration reflects the relative abundance of each feature under the two experimental conditions. Molecular families were annotated through GNPS spectral library matches and compound class predictions obtained via SIRIUS. In ESI­(+), predominant families include betaine lipids, glycerophospholipids, fatty amides, carnitine derivatives, siderophores, and small peptides. In ESI(−), major families comprise phenolic acids, lysophosphatidylethanolamines, glycerophospholipids, small peptides, aminoglycosides, and oxylipins.

To further validate the statistical findings, annotated features that displayed differences in production according to the statistical analysis were selected. Among these, only six features were identified. For these six metabolites, extracted ion chromatograms were generated, and the corresponding peak areas were manually integrated. A detailed analysis of these six features is presented in Figure 6. Among them, the hydroxamate siderophore ferrichrome C (*m*/*z* 755.2531; RT 2.54 min) displayed a marked increase in peak area under hematite treatment, indicating the induction of its production in the presence of the mineral (Figure 6A). Similarly, phosphatidic acid 16:0/18:2 (*m*/*z* 671.4656; RT 7.91 min) was detected exclusively in treated samples (Figure 6B).

**Figure 6:**
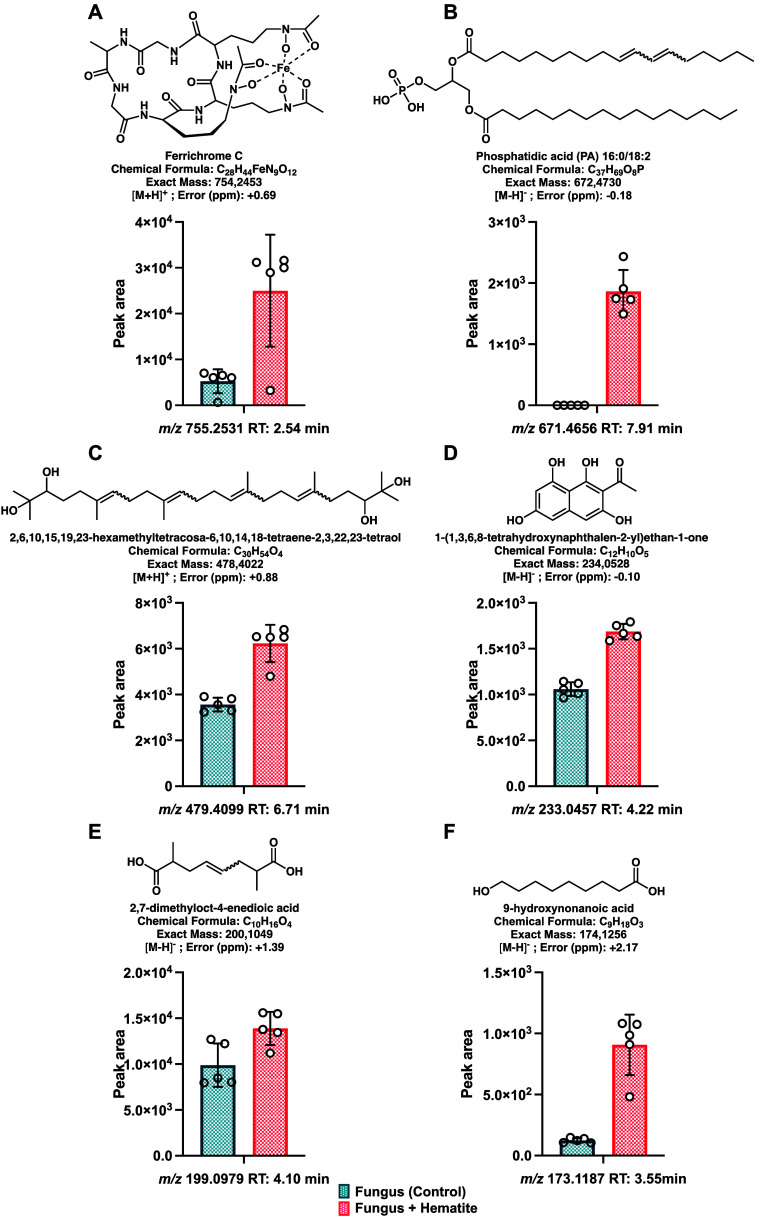
Effect of hematite exposure on six annotated metabolites in *R. similis* LaBioMMi 1217 detected by untargeted UHPLC–MS/MS. Panels present peak area comparisons between treatments for metabolites under both ionization modes, all with significantly higher abundance in hematite-exposed cultures (*p* < 0.05, mass error <5 ppm).

2,6,10,15,19,23-hexamethyltetracosa-6,10,14,18-tetraene-2,3,22,23-tetraol (*m*/*z* 479.4099; RT 6.71 min) also exhibited an increase in intensity upon hematite addition, suggesting the possible involvement of polyhydroxylated terpenoids in the differential metabolism (Figure 6C). Among the polyketides related to the melanin pathway, 1-(1,3,6,8-tetrahydroxynaphthalen-2-yl)­ethan-1-one (*m*/*z* 233.0457; RT 4.22 min) featured a higher relative abundance in the treated condition (Figure 6D). 2,7-Dimethyloct-4-enedioic acid (*m*/*z* 199.0979; RT 4.10 min) displayed a more discrete increase under hematite treatment (Figure 6E). Finally, 9-hydroxynonanoic acid (*m*/*z* 173.1187; RT 3.55 min) was detected at low intensity in the control condition but at a markedly higher level in the mineral-treated condition (Figure 6F).

Overall, all six metabolites displayed significantly higher intensities in the presence of hematite, with some compounds being absent or near the detection limit under the control condition. These results indicate that the fungus–mineral interaction selectively influences the production of siderophores, lipids, terpenoids, and polyketide derivatives, potentially reflecting metabolic adaptations to an iron-enriched microenvironment.

Taken together, we demonstrate that hematite exposure promotes a consistent increase in the levels of metabolites related to siderophore biosynthesis, lipid remodeling, terpenoid pathways, and polyketide-derived metabolism. The FBMN results complemented these findings, revealing that the mineral affects multiple metabolic domains in *R. similis* LaBioMMi 1217, with marked changes in membrane lipid composition, iron-chelating compounds, and other specialized metabolites. Mirror plots comparing the experimental spectra with GNPS reference spectra and the annotations performed using SIRIUS for all identified metabolites are provided in the Supporting Information.

## Discussion

### Role of Black Fungi in Mineral Dissolution

Microorganisms play key roles in mineral dissolution and transformation, with archaea and bacteria, particularly acidophilic species such as *Acidithiobacillus ferrooxidans*, *Leptospirillum ferrooxidans*, and *Ferroplasma acidarmanus*, being well-studied for their capacity to oxidize metals in extreme environments.^37,38^ Although historical research primarily focused on prokaryotes, fungi are also capable of promoting mineral dissolution and metal mobilization. On their own, fungi such as *Aspergillus niger* and *Penicillium simplicissimum* are well-known for their ability to leach metals such as iron (Fe), manganese (Mn), and aluminum (Al) from mineral substrates, whereas black yeasts and lichenized fungi contribute to the degradation of rock surfaces in natural environments.^39,40^ In iron-rich minerals such as hematite, these processes are particularly relevant because of the low solubility and high thermodynamic stability of Fe^3+^ ions under neutral pH.^41,42^

In this study, we focused on the interaction of the black fungus *R. similis* LaBioMMi 1217 with hematite, in consideration of its polyextremophilic nature, as previous studies demonstrated that this fungus can survive in synthetic regolith designed to simulate the geochemical composition of Mars while also producing secondary metabolites potentially associated with iron metabolism.^30^

The Fe^2+^ ion levels progressively increased over time exclusively in the iron oxide-rich condition. This pattern indicates that the fungus promoted the reduction of insoluble Fe­(III) present in iron oxides to more soluble Fe­(II), in line with active reductive processes previously described in fungus–mineral interactions.^43^ In parallel, the acidification observed was consistent with the secretion of low-molecular-weight organic acids, a process characteristic of several fungi, including *Aspergillus* species, which can acidify the medium through citric acid production.^44^ A similar mechanism might have occurred in this study, contributing to the dissolution of the mineral surface and facilitating iron mobilization.^45^

The hematite surface exhibited distinct morphological modifications after fungal interaction, including microfractures and localized surface wear, which were absent in the control. Furthermore, fungal structures (i.e., filamentous hyphae and yeast-like cells) directly adsorbed onto the hematite surface, forming a biofilm. This close physical association suggests that extracellular components within the biofilm help promote localized mineral dissolution and surface corrosion, as reported in previous bioweathering studies.^46,47^

### Molecular Strategies and Chemical Mechanisms of *R. similis* LaBioMMi 1217 in Hematite Dissolution

The genomic organization identified in *R. similis* LaBioMMi 1217 revealed a complete set of genes involved in the biosynthesis of ferrichrome-type siderophores, including *sidA*, *sidC*, *sidD*, *sidF*, *sidH*, *sidI*, and *sidL*. These genes are distributed across two contigs, and they retain the functional architecture described in other filamentous fungi, indicating conservation of the biosynthetic pathway.^26,48^ The central role of *sidA* in the N^5^-hydroxylation of ornithine, followed by the coordinated action of *sidF* and *sidC* to produce N^5^-acetyl-N^5^- hydroxyornithine and assemble the cyclopeptidic core, is consistent with the classical ferrichrome synthesis mechanism. The presence of *sidH* and *sidL* indicates enzymatic steps required for the maturation of fusarinine-type siderophores involving specific reactions such as the dehydroxylation of mevalonyl-CoA.^49^ This arrangement reflects the metabolic adaptability of melanized fungi in producing different variants of the siderophores.

LC–MS/MS-based metabolomic data corroborated the functional expression of this pathway, with ferrichrome C detected in higher abundance in cultures exposed to hematite, suggesting the induction of siderophore biosynthesis in response to the mineral. The identification of the transporters STR1 and ARN1 further supports the existence of an active siderophore export and reuptake system, similar to what is described in *A. fumigatus* and *Fusarium graminearum*.^50,51^ This capability provides an adaptive advantage in niches with low iron bioavailability, such as oligotrophic soils and ferric oxide-rich environments. From an astrobiological perspective, this metabolism is particularly relevant, as iron mobilization from poorly soluble minerals represents a plausible survival strategy in Mars analog environments, where iron is abundant but predominantly present in the insoluble ferric form.^52^

Beyond their primary roles in iron chelation and transport, siderophores might also indirectly participate in the reduction of Fe^3+^ to Fe^2+^, thereby influencing redox dynamics in mineral microenvironments. After Fe^3+^ complexation, internalization of the siderophore–iron complex is often followed by intracellular reduction reactions catalyzed by specific reductases, releasing Fe^2+^ for metabolic use.^49,53^ Although the intracellular pathway was inferred for *R. similis* LaBioMMi 1217, iron reduction can also occur extracellularly, suggesting complementary mechanisms that extend beyond internal metabolic processes. Redox-active metabolites derived from the melanin biosynthetic pathway, such as the aromatic polyketides annotated [e.g., 1-(1,3,6,8-tetrahydroxynaphthalen-2-yl)­ethan-1-one, 7-acetyl-3,5,6,8-tetrahydroxy-3,4,5,8-tetrahydronaphthalen-1-one, 3-acetyl-2,5,7-trihydroxynaphthalene-1,4-dione], one of which was predominantly produced in the presence of hematite, can act as electron transfer mediators, promoting the direct reduction of Fe^3+^ in hematite.

This mechanism is favored under acidic pH, consistent with the observed increase in organic acid production under the hematite-exposed condition. The accumulation of carboxylic acids detected in the metabolomic data set directly supports the first step of the model, indicating active extracellular acidification by the fungus. This process is likely accompanied by the generation of reactive oxygen species (ROS), such as hydroxyl radicals (•OH), which can exert oxidative stress on cellular components, particularly lipids and cell wall structures. In this context, the detection of oxidized squalene derivatives (e.g., 2,6,10,15,19,23-hexamethyltetracosa-6,10,14,18-tetraene-2,3,22,23-tetraol) strongly suggests a protective role, as squalene is a well-known antioxidant capable of scavenging free radicals and reactive oxygen intermediates. Thus, siderophore biosynthesis, in synergy with secondary metabolism associated with melanization, constitutes an integrated system for iron acquisition and mineral modification, with direct implications for the nutrient mobilization and geochemical alteration of substrates. A mechanistic proposal for this scenario is illustrated in Figure 7.

**Figure 7:**
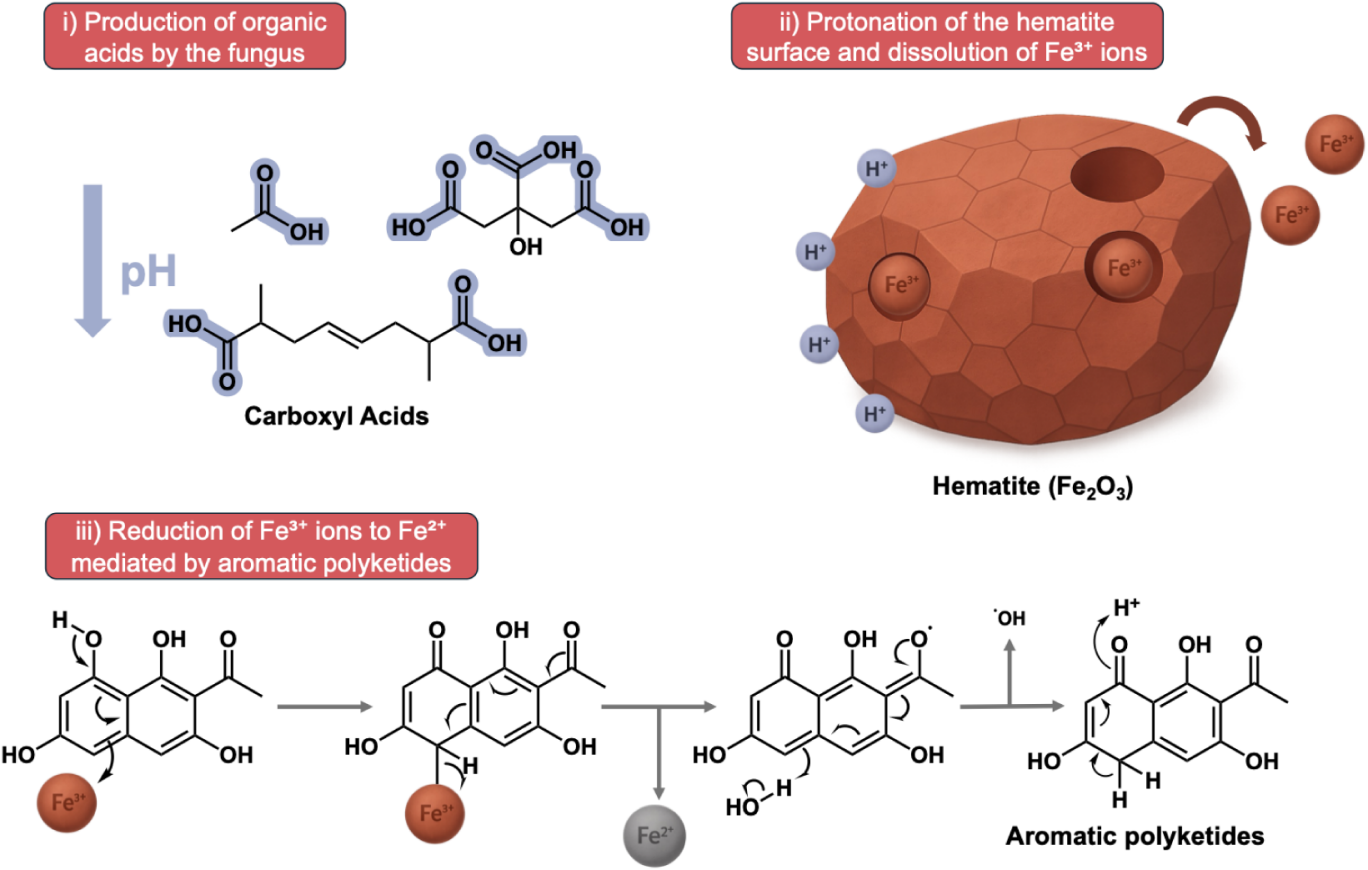
Mechanistic pathway for fungus-mediated hematite dissolution and iron redox transformation. The scheme summarizes the proposed sequence of processes in which organic acids promote local acidification, facilitating hematite surface protonation and Fe^3+^ release, while aromatic polyketides mediate the subsequent reduction to Fe^2+^. Together, these metabolic activities integrate chemical dissolution, redox transformation, and mineral surface modification by the fungus.

### Astrobiological Implications for the Search for Life on Mars

In this study, we demonstrated that the black fungus *R. similis* LaBioMMi 1217, which is resistant to Mg-perchlorate and UV–C irradiation,^30,54^ could modify the structure and chemical composition of hematite through combined mechanisms of acidification, chelation, and reduction, with clear evidence of surface corrosion and Fe^2+^ ion mobilization. On Mars, analogous processes might occur in transiently wet microenvironments, such as subsurface brines or thin water films adsorbed onto hygroscopic minerals, in which microorganisms adapted to low water activity, high salinity, and oxidative stress could sustain metabolic activity. Moreover, similar biogeochemical interactions might have been even more widespread in Mars’ past during periods when liquid water was stable on the surface, and riverine, lacustrine, and deltaic systems provided more favorable conditions for sustained microbial colonization and mineral alteration.^28,55^

Hydroxamate siderophores (e.g., ferrichrome C), melanin pathway polyketides, lipids, and carotenoids, each characterized by high chemical stability^56,57^ and increased levels under iron-rich conditions, have emerged as promising priority targets for life-detection instrumentation. This is particularly relevant in the context of upcoming planetary missions, such as the ExoMars mission, whose Mars Organic Molecule Analyzer includes mass spectrometry capabilities designed to detect and characterize such stable organic compounds directly on the Martian surface and subsurface through the analysis of material collected by drilling, also planned as part of the mission strategy.^55,58^ Furthermore, the ability of *R. similis* LaBioMMi 1217 to induce detectable crystallographic alterations in hematite suggests that such modifications could be investigated on Mars using diffraction and spectroscopic techniques already deployed or proposed for *in situ* missions.^59^

## Conclusion

This study significantly advances our understanding of secondary metabolism in black fungi under Mars analogue conditions, showing that *R. similis* LaBioMMi 1217 mobilizes iron through coordinated mechanisms of acidification, chelation, and reduction. Metabolomic analyses revealed the production of siderophores, aromatic polyketides, and other stable metabolites, the synthesis of which was intensified in the presence of hematite, indicating an adaptive metabolic response directly linked to interactions with iron-rich minerals. These findings reinforce the role of metabolomics as a powerful tool for astrobiology, capable of identifying potential chemical biosignatures that could be preserved and detected in extraterrestrial environments.

The integration of mineralogical, genomic, and metabolomic data further demonstrated that *R. similis* LaBioMMi 1217 mobilizes iron through a coordinated suite of chemical and metabolic pathways, resulting in detectable modifications to the hematite crystal structure and the production of stable, characterizable organic compounds. These two complementary lines of evidence, namely mineralogical and chemical biosignatures, provide a practical framework for designing life-detection protocols on Mars. The inclusion of extremophilic eukaryotes in models of potential Martian ecology broadens the range of target metabolites and suggests that the diversity of microbe–mineral interactions might be greater than traditionally considered. By combining multiple adaptive strategies, *R. similis* LaBioMMi 1217 emerged as a relevant model organism for testing hypotheses on the persistence and detectability of life in extreme planetary environments.

## Supplementary Material







## Data Availability

The annotated genome sequence of *Rhinocladiella similis* LaBioMMi 1217 has been deposited in the GenBank database under accession number JAZDCV000000000 (BioProject PRJNA1068593). The GNPS workflows for LC–MS/MS data acquired in positive and negative ionization modes are accessible at: https://gnps2.org/status?task=cd2de24cc4af448a90646c70eace9843e and https://gnps2.org/status?task=a05823c0985f4a10a26468ff27022d15. Additional data supporting the findings of this study are available from the corresponding author upon reasonable request.

## Notes

Supplementary Data 1 – Significant TBLASTN alignments of siderophore biosynthesis genes and general iron metabolism genes in *Rhinocladiella similis* LaBioMMi 1217 (XLSX)
Supplementary Data 2 – Full feature list; positive/negative ESI modes; ion formulas; retention times; fold changes; statistical significance (XLSX)
Growth curves; Fe^2+^ calibration curve; metabolomics workflow; MZmine parameters; iron-related genes; UHPLC–MS/MS annotations; experimental and predicted MS/MS spectra; spectral matches; fragmentation schemes (PDF)
