# Supplementary material for: Fungal–Mineral Interaction: Astrobiology Insights from Iron-Rich Mineral Alteration by an Extremophile Black Fungus
Source: JACS Au. 2025 Dec 15;6(1):446–59. doi: 10.1021/jacsau.5c01365 (PMC12848704; doi:10.1021/jacsau.5c01365)
Supplement: Supplementary file 3 [file au5c01365_si_003.pdf]

Supplementary Material

Fungal-Mineral Interaction: Astrobiology Insights from Iron-Rich Mineral Alteration by an Extremophile Black Fungus

Alef dos Santos[1,2,3*], Fluvio Molodon[4,5], Júnia Schultz[6], Mauricio Augusto P. M. da Silva Alves[1], Alexandre Soares Rosado[6], Kurt Konhauser[2], Edson Rodrigues-Filho[1], and Merve Yeşilbaş[3*]

[1]Department of Chemistry, Federal University of São Carlos, São Carlos, 13565-905, Brazil
[2]Department of Earth & Atmospheric Sciences, University of Alberta, Edmonton, T6G 2E3, Canada
[3]Department of Chemistry, Umeå University, Umeå, SE-90187, Sweden
[4]Department of Ecology, Environment and Geoscience, Umeå University, Umeå, SE-90187, Sweden
[5]Oceanographic Institute, University of São Paulo, São Paulo, 05508-020, Brazil
[6]Biological and Environmental Science and Engineering Division, King Abdullah University of Science and Technology, Thuwal, 23955, Saudi Arabia

*Corresponding Authors
Alef dos Santos
alef.ds@umu.se

Merve Yeşilbaş
merve.yesilbas@umu.se

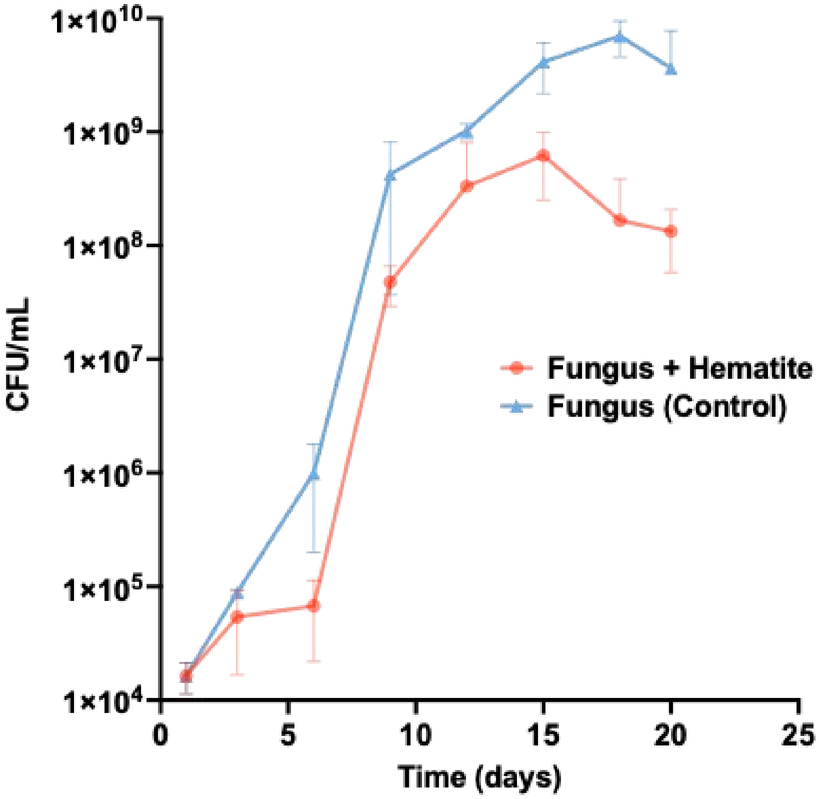

Figure S1. Growth curves by CFU of *Rhinocladiella similis* LaBioMMi 1217 under control and treatment conditions. Cell growth was monitored over time and expressed as the mean ± standard deviation (n = 3). The red line represents the treatment condition, and the blue line represents the control condition.

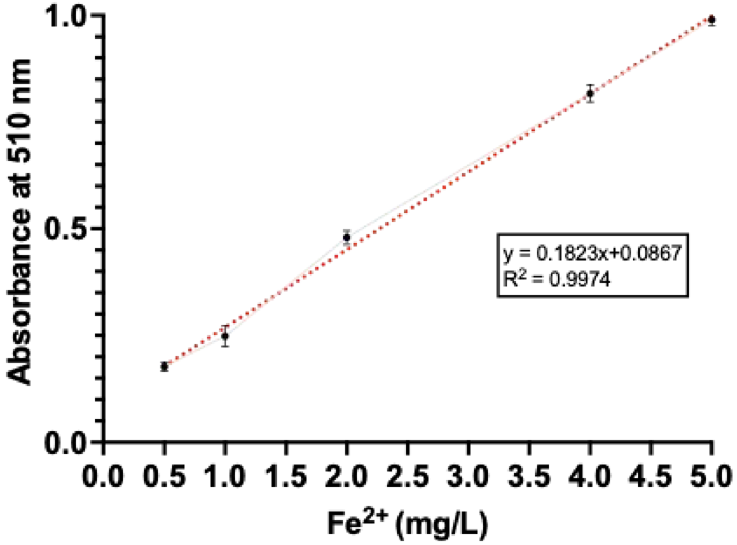

Figure S2. Calibration curve for ferrous ion (Fe^{2+}) quantification using the 1,10-phenanthroline complexation method. The curve was constructed from $FeSO_4$ standard solutions (0.5–5.0 mg/L) with three replicates per concentration (*n* = 3) and fitted by linear regression (red dotted line). The coefficient of determination (R^2) confirmed the suitability of the model for quantitative analysis at 510 nm using a 1-cm path length cuvette.

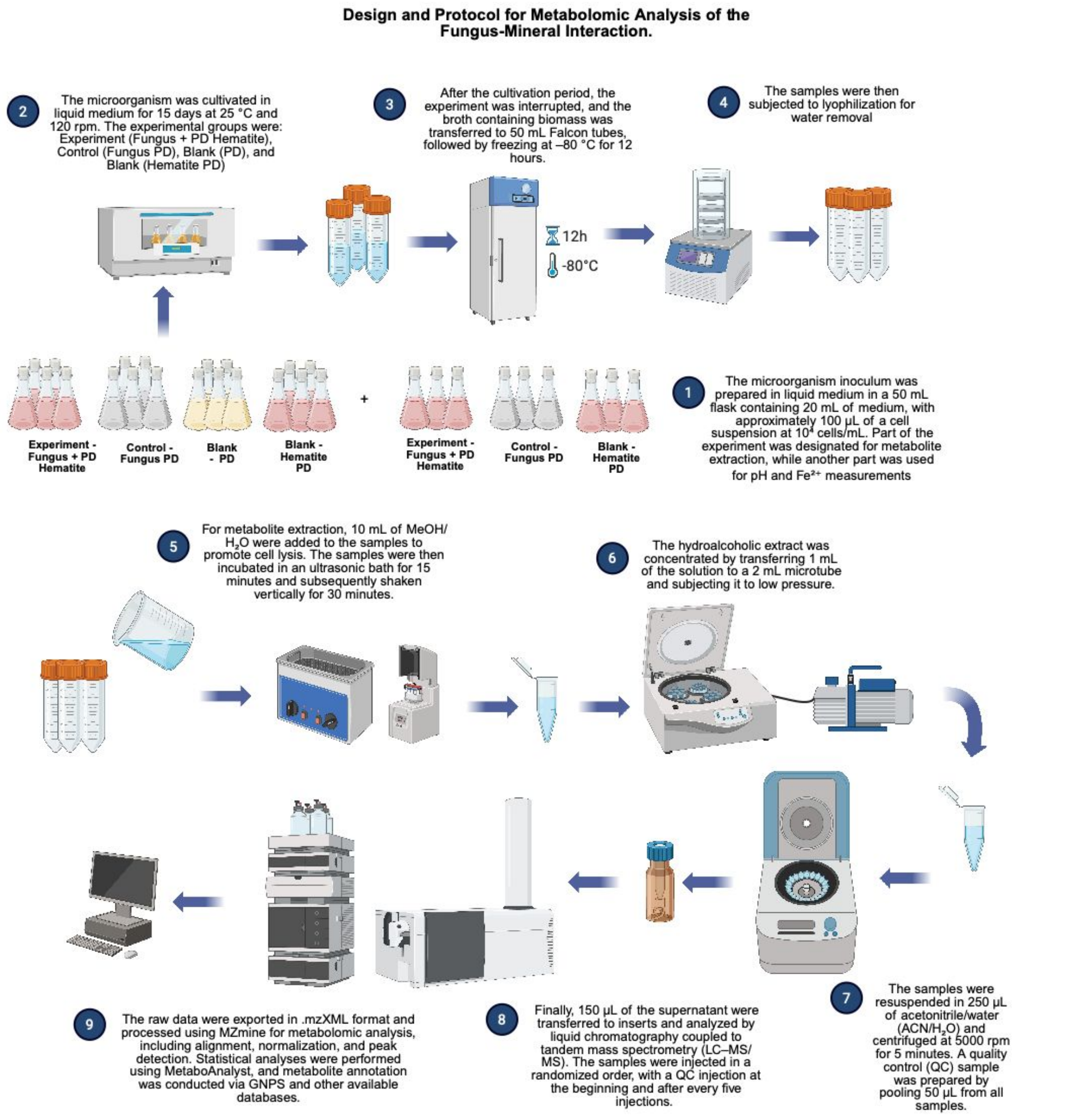

Figure S3. Experimental design and workflow for metabolomic analysis of the *Rhinocladiella similis*–hematite interaction.

Table S1. MZmine processing parameters for liquid chromatography–tandem mass spectrometry data analysis

Processing step	Parameter/setting
Data conversion	Raw files converted to .mzML using MSConvert
Mass detection	MS1 threshold: 1.00×10^3; MS2 threshold: 1.00×10^1; MS tolerance: 10 ppm
Smoothing	Applied
Chromatogram building	ADAP chromatogram builder; *m/z* tolerance: 0.02 Da; minimum time span: 0.05 min
Chromatogram deconvolution	Local minimum resolver (90%, 0.1 min, 1%, 1.00×10^3, 2.00, 0.01–5 min, four scans)
Isotope filtering	5 ppm, 0.05 min, 2
Feature alignment	Join aligner; 10 ppm, 0.3 min, 3:1
Gap filling	20%
Feature filtering	Min peaks in a row: 2
Duplicate filtering	Yes
Blank filtering	Features were removed if detected in ≥8 blank samples (hematite + PDB medium blanks, $n = 5$; PDB medium blanks, $n = 5$) and if their peak area in experimental samples was less than 4-fold higher than that in blanks (FC < 4).
Polarity-specific adjustments	For ESI(−): MS1 threshold = 1.00×10^2; MS2 threshold = 1.00; MS tolerance = 10 ppm

PDB: potato dextrose broth; ESI: electrospray ionization

Table S2. Fungal iron metabolism-related genes identified in the genome of *Rhinocladiella similis* LaBioMMi 1217 with >40% sequence identity to reference sequences

Gene	Main function	Fungal reference organism	No. of copies (>40% identity)
ARN1	High-affinity siderophore transporter (ferric enterobactin transporter)	*Saccharomyces cerevisiae*	1
CCC1	Vacuolar iron transporter; intracellular iron homeostasis	*Saccharomyces cerevisiae*	3
FET3	Multicopper ferroxidase (oxidizes Fe^{2+} to Fe^{3+} for iron uptake)	*Saccharomyces cerevisiae*	12
FETC	Permease component of siderophore-dependent iron transport system	*Aspergillus fumigatus*	18
FTR1	High-affinity Fe^{3+} permease (associated with FET3)	*Candida albicans*	2
FTRA	FTR1-like permease (putative role in Fe^{3+} transport)	*Candida albicans*	2
SEF1	Transcription factor regulating iron uptake genes	*Candida albicans*	1
SFU1	Repressor of iron acquisition genes under high-iron conditions	*Candida albicans*	6
STR1	Siderophore transporter of the ferric-hydroxamate type	*Aspergillus nidulans*	2

Table S3. Liquid chromatography–tandem mass spectrometry-based annotation of major metabolites in methanolic extracts from *Rhinocladiella similis.* Annotations were performed according to precursor mass values, cross-referenced with GNPS spectral libraries, *in silico* predictions using SIRIUS, and manual annotation supported by literature data.

No.	Annotation	Retention time (min)	Adduct	*m/z*	Molecular formula	Error (ppm)
**Betaine lipids**						
**1**	DGTS(0:0/18:0)	6.91	$[M+H]^+$	502.41077	$C_{28}H_{55}NO_6$	1.1
**2**	DGTSA(18:1/18:2)	7.88	$[M+H]^+$	762.625	$C_{46}H_{83}NO_7$	1.01
**Carboxylic acids**						
**3**	2-Isopropylmalic acid	2.61	$[M-H]^-$	175.06179	$C_7H_{12}O_5$	3.39
**4**	Malic acid	0.40	$[M-H]^-$	133.01525	$C_4H_6O_5$	7.54
**5**	2,7-Dimethyloct-4-enedioic acid	4.10	$[M-H]^-$	199.09791	$C_{10}H_{16}O_4$	1.64
**6**	9-Hydroxynonanoic acid	3.55	$[M-H]^-$	173.1187	$C_9H_{18}O_3$	2.17
**Carnitine derivatives**						
**7**	Butyrylcarnitine	1.83	$[M+H]^+$	232.15476	$C_{11}H_{21}NO_4$	1.83
**8**	Carnitine	1.00	$[M+H]^+$	162.12049	$C_7H_{15}NO_3$	49.47
**Glycerophospholipids**						
**9**	LPA 18:1	7.39	$[M-H]^-$	435.2519	$C_{21}H_{41}O_7P$	1.69
**10**	LPA 18:1	7.26	$[M-H]^-$	435.25242	$C_{21}H_{41}O_7P$	2.88
**11**	LPC 18:1	6.49	$[M+H]^+$	522.35728	$C_{26}H_{52}NO_7P$	3.57
**12**	LPC 18:2	6.19	$[M+formate]^-$	564.33143	$C_{26}H_{50}NO_7P$	1.31
**13**	Hexosyl LPE 16:0	6.34	$[M+H]^+$	616.34512	$C_{27}H_{54}NO_{12}P$	−0.84
**14**	LPE 16:0	6.34	$[M-H]^-$	452.27834	$C_{21}H_{44}NO_7P$	1.38
**15**	LPE 16:0	6.44	$[M-H]^-$	452.27873	$C_{21}H_{44}NO_7P$	2.24
**16**	LPE 18:2	6.04	$[M-H]^-$	476.27804	$C_{23}H_{44}NO_7P$	0.68
**17**	LPS 18:1	6.78	$[M-H]^-$	522.2841	$C_{24}H_{46}NO_9P$	1.73
**18**	LPS 18:1	6.66	$[M-H]^-$	522.28371	$C_{24}H_{46}NO_9P$	0.99

**19**	PA(16:0/18:2)	7.91	$[M-H]^-$	671.4656	$C_{37}H_{69}O_8P$	0.18
**20**	PA(16:1/18:2)	9.43	$[M-H]^-$	669.4499	$C_{37}H_{67}O_8P$	0.55
**21**	PA(18:2/18:2)	9.45	$[M-H]^-$	695.46541	$C_{39}H_{69}O_8P$	0.33
**22**	PC(16:0/18:1)	8.88	$[M+H]^+$	760.5869	$C_{42}H_{82}NO_8P$	2.39
**23**	PC(16:0/18:3)	8.15	$[M+H]^+$	756.55455	$C_{42}H_{78}NO_8P$	1.02
**24**	PC(18:2/18:2)	8.01	$[M+H]^+$	782.57088	$C_{44}H_{80}NO_8P$	1.85
**25**	PC(O-18:0/20:4)	7.31	$[M+H]^+$	796.62933	$C_{46}H_{86}NO_7P$	9.87
**26**	PE(18:18:1)	8.77	$[M+H]^+$	744.55421	$C_{41}H_{78}NO_8P$	0.57
**27**	PI(34:2)	9.55	$[M-H]^-$	833.51829	$C_{43}H_{79}O_{13}P$	0.34
**28**	PS(16:0/18:2)	9.08	$[M+H]^+$	760.51224	$C_{40}H_{74}NO_{10}P$	−0.09
**Oxylipins[(a)]**						
**29**	12,13-DiHOME	5.67	$[M-H]^-$	313.23855	$C_{18}H_{34}O_4$	2.12
**30**	9(S)-HOTrE	5.29	$[M+H-H_2O]^+$	277.21631	$C_{18}H_{30}O_3$	0.37
**Phenolic acids and derivatives**						
**31**	3-Hydroxycinnamic acid	3.63	$[M-H]^-$	163.04074	$C_9H_8O_3$	7.49
**32**	5,7-Dimethoxychroman-2-one	3.00	$[M-H]^-$	207.06662	$C_{11}H_{12}O_4$	2.22
**33**	5-Hydroxyindoleacetic acid	3.15	$[M-H]^-$	190.05114	$C_{10}H_9NO_3$	3.8
**Siderophores[(b)]**						
**34**	Desferri-ferricrocin	2.41	$[M+H]^+$	718.33658	$C_{28}H_{47}N_9O_{13}$	−0.04
**35**	Ferrichrome C	2.54	$[M+H]^+$	755.2531	$C_{28}H_{44}FeN_9O_{12}$	−0.08
**36**	Ferricrocin	2.43	$[M+H]^+$	771.24861	$C_{28}H_{44}FeN_9O_{13}$	0.7
**37**	Desferri-ferricrocin	2.41	$[M-H]^-$	716.322	$C_{28}H_{47}N_9O_{13}$	−0.08
**Small peptides**						
**38**	(3-Carboxybutanoyl)proline	2.11	$[M+H-H_2O]^+$	212.09191	$C_{10}H_{15}NO_5$	3.67
**39**	(5-Oxopyrrolidine-2-carbonyl)proline	1.78	$[M+H]^+$	227.10308	$C_{10}H_{14}N_2O_4$	1.97
**40**	(2,1-Carboxy-2-methylbutyl)amino)propane-1,2,3-tricarboxylic acid	2.93	$[M+H]^+$	306.11857	$C_{12}H_{19}NO_8$	0.74
**41**	Isoleucine	1.06	$[M+H]^+$	132.10228	$C_6H_{13}NO_2$	2.84
**Fatty amides**						

**42**	14-Oxo-14-(pyrrolidin-1-yl)tetradecanoic acid	4.33	$[M+H]^+$	343.29586	$C_{19}H_{38}N_2O_3$	0.99
**Carotenoids**						
**43**	,6,10,15,19,23-hexamethyltetracosa-6,10,14,18-tetraene-2,3,22,23-tetraol	6.71	$[M+H]^+$	479.409	$C_{30}H_{54}O_4$	+0.88
**Nucleosides**						
**44**	Isopentenyladenosine	3.6	$[M+H]^+$	336.16657	$C_{15}H_{21}N_5O_4$	−0.18
**Aromatic polyketides[c,d]**						
**45**	1-(1,3,6,8-Tetrahydroxynaphthalen-2-yl)ethan-1-one	4.22	$[M-H]^-$	233.0457	$C_{12}H_{10}O_5$	−0.10
**46**	7-Acetyl-3,5,6,8-tetrahydroxy-3,4,5,8-tetrahydronaphthalen-1-one	3.7	$[M-H]^-$	235.0616	$C_{12}H_{12}O_5$	−0.41
**47**	3-Acetyl-2,5,7-trihydroxynaphthalene-1,4-dione	3.3	$[M-H]^-$	247.0254	$C_{12}H_8O_6$	0.36

(a) Compounds manually annotated by comparison with data reported by Almeida-Trapp et al. (2021) — Development, validation, and application of an HPLC-MS/MS method for quantification of oxidized fatty acids in plants. Journal of Chromatography B, 1186, 123006. https://doi.org/10.1016/J.jchromb.2021.123006 **(b)** Compounds manually annotated by comparison with data presented by dos Santos et al. (2024) — Rhinocladiella similis: A Model Eukaryotic Organism for Astrobiological Studies on Microbial Interactions with Martian Soil Analogs. JACS Au, 5, 203. https://doi.org/10.1021/jacsau.4c00869 **(c,d)** Compounds manually annotated by comparison with data reported by Gao et al. (2022) — Fungal Melanin Biosynthesis Pathway as Source for Fungal Toxins. mBio, 13(3), e00219-22. https://doi.org/10.1128/mbio.00219-22.

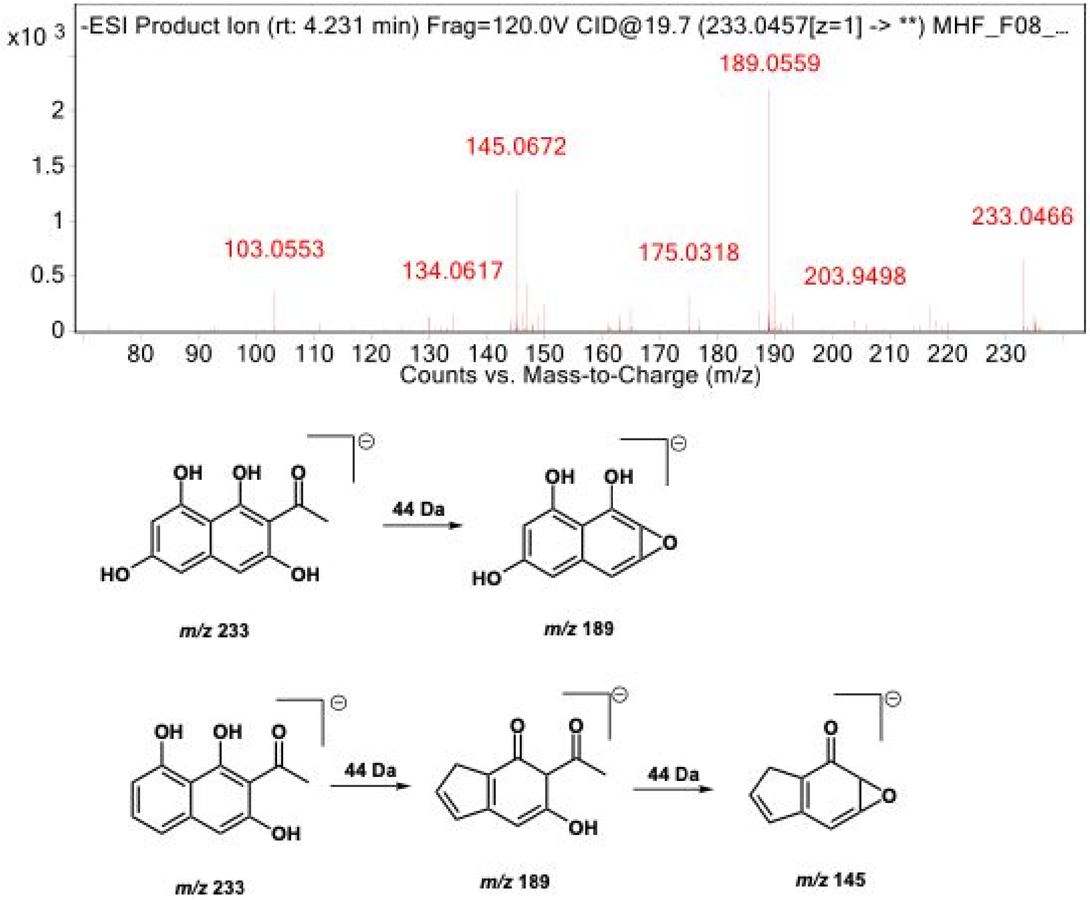

Figure S4. Experimental tandem mass spectrometry spectrum and proposed identity for the feature at *m/z* 233.0457 ([M–H]⁻, RT 4.231 min). Data were acquired in the negative electrospray ionization mode with CID. Major product ions are observed at *m/z* 103.0553, 134.0617, 145.0672, 175.0318, 189.0559, 203.9498, and 233.0466. Proposed fragmentation pathway for the feature at *m/z* 233.0445 ([M–H]⁻) annotated as 1-(1,3,6,8-tetrahydroxynaphthalen-2-yl)ethan-1-one ($C_{12}H_{10}O_5$). This scheme presents a proposed fragmentation

pathway, presenting sequential neutral losses of 44 Da from the precursor ion (*m/z* 233) to generate fragment ions at *m/z* 189 and subsequently at *m/z* 145.

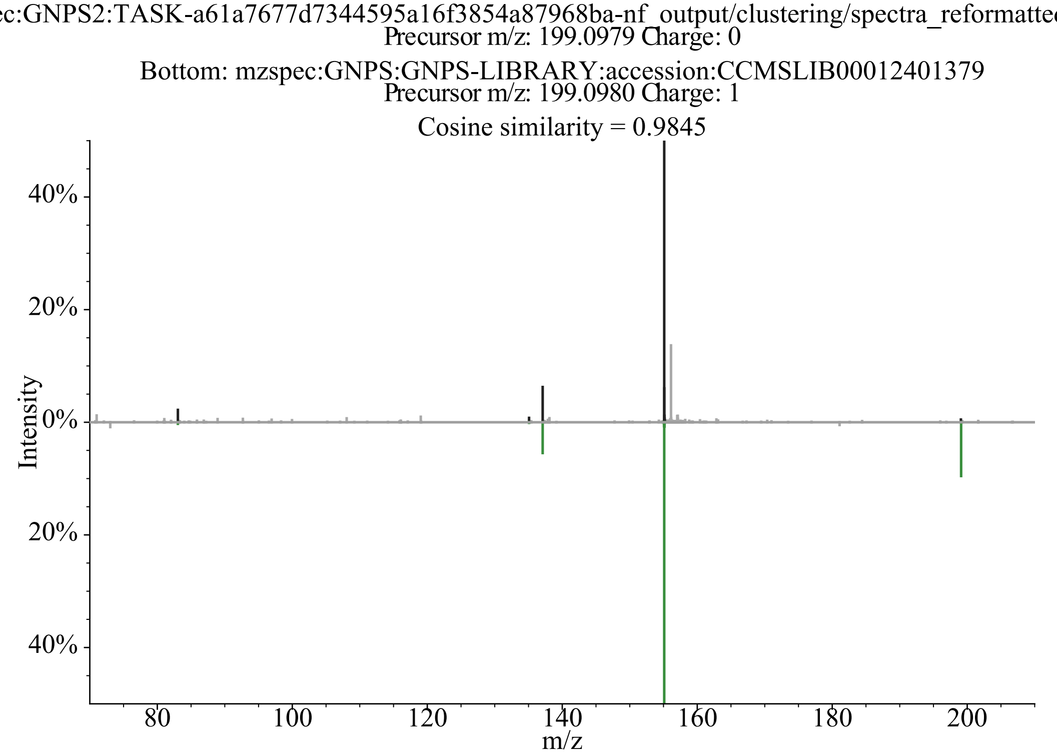

Figure S5. Tandem mass spectrometry (MS/MS) spectral match for the feature annotated as 2,7-dimethyloct-4-enedioic acid ($[M-H]^-$, *m/z* 199.09791,$C_{10}H_{16}O_4$). The top spectrum corresponds to the experimental MS/MS data acquired the in negative ionization mode, and the bottom spectrum represents the reference spectrum from the Global Natural Products Social Molecular Networking library (accession CCMSLIB00012401379). The match exhibited a cosine similarity score of 0.9845.

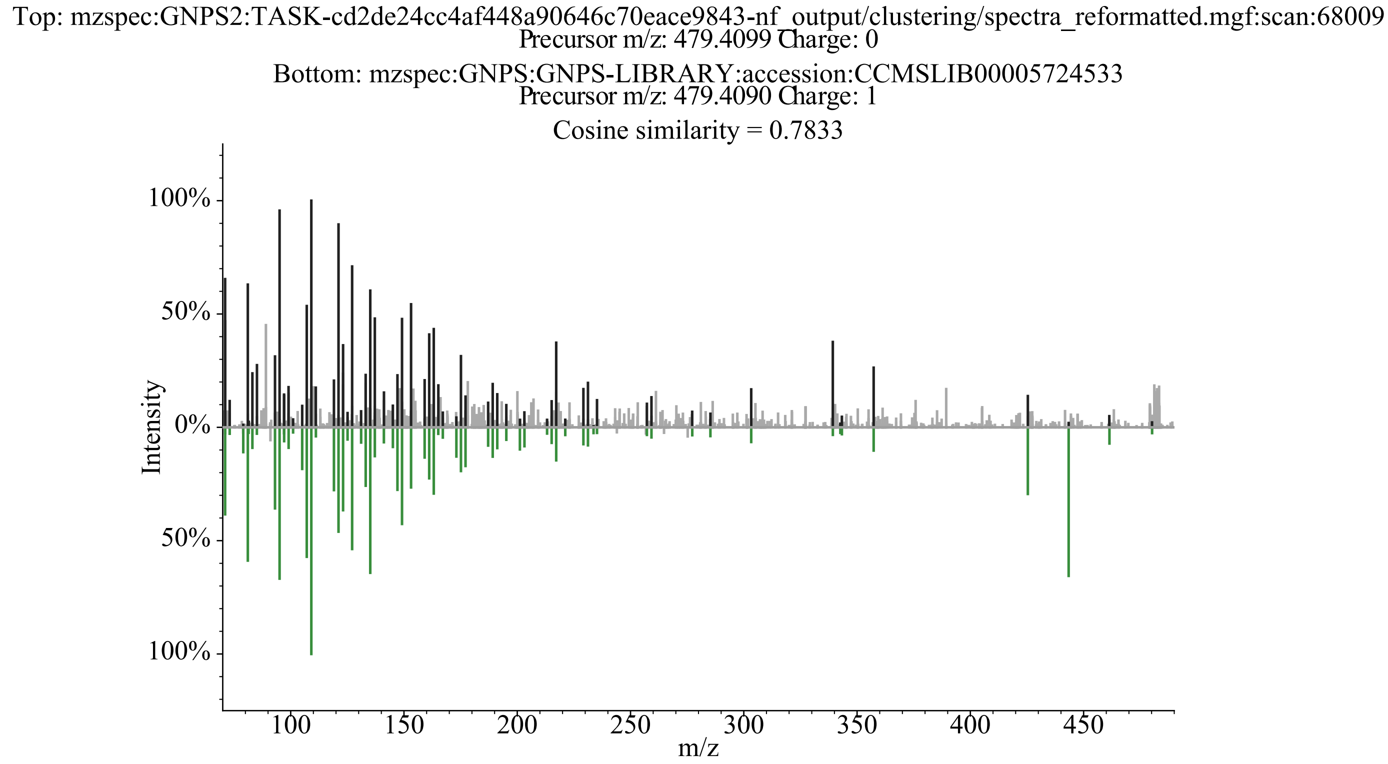

Figure S6. Tandem mass spectrometry (MS/MS) spectral match for the feature annotated as ,6,10,15,19,23-hexamethyltetracosa-6,10,14,18-tetraene-2,3,22,23-tetraol ([M+H]$^+$, *m/z* 479.409, $C_{30}H_{54}O_4$, retention time 6.71 min). The top spectrum corresponds to the experimental MS/MS data acquired in positive ionization mode, and the bottom spectrum represents the reference spectrum from the Global Natural Products Social Molecular Networking library (accession CCMSLIB00005724533). The match exhibited a cosine similarity score of 0.7833.

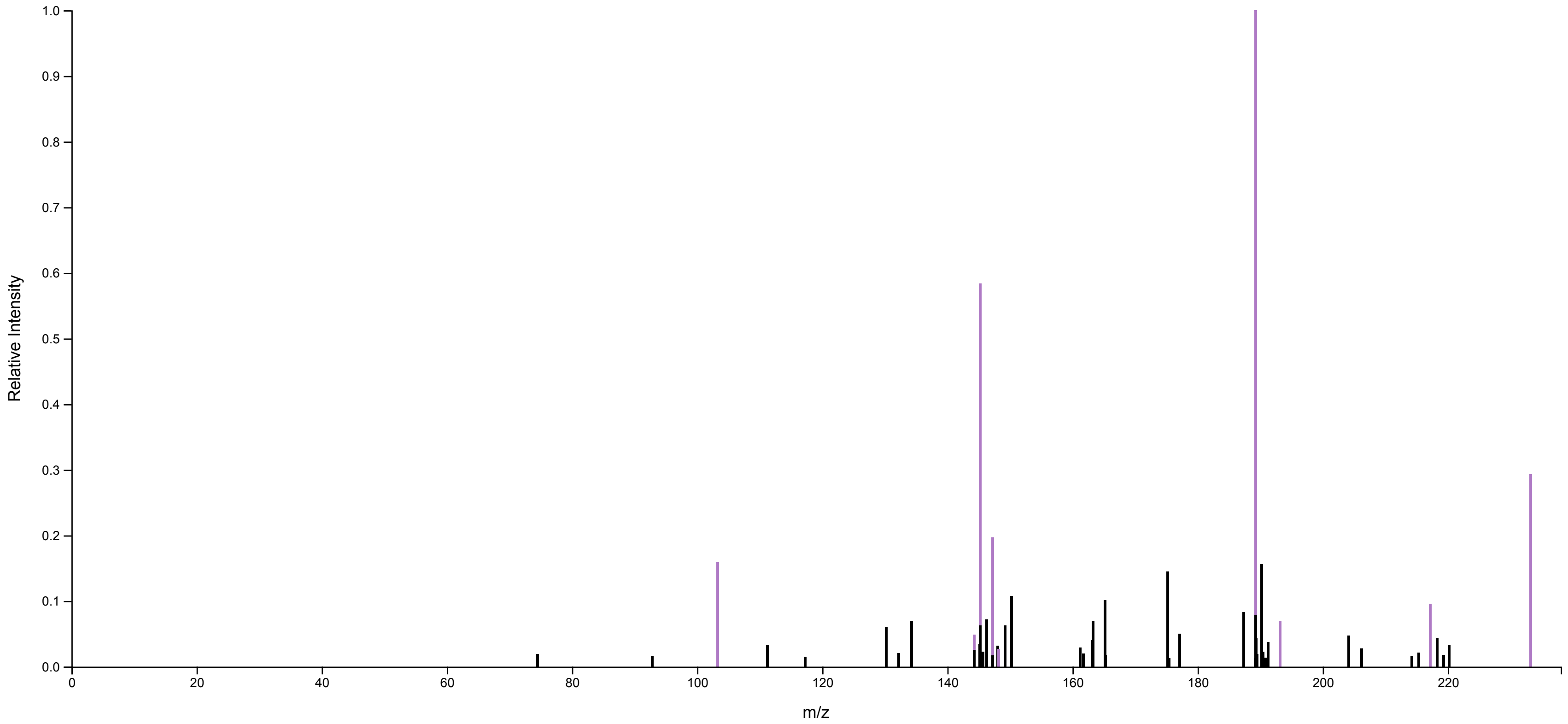

Figure S7. Tandem mass spectrometry (MS/MS) spectral prediction for the feature annotated as 1-(1,3,6,8-tetrahydroxynaphthalen-2-yl)ethan-1-one ($[M–H]^-$, *m/z* 233.0445,$C_{12}H_{10}O_5$). The black trace represents the experimental MS/MS spectrum acquired in the negative ionization mode, whereas the purple trace corresponds to the *in silico* fragmentation prediction generated by SIRIUS. The predicted structure was assigned with 54.13% confidence (CSI:FingerID score: −144,950; XLogP: 2.50), and it is indexed in COCONUT and PubChem as a natural product.

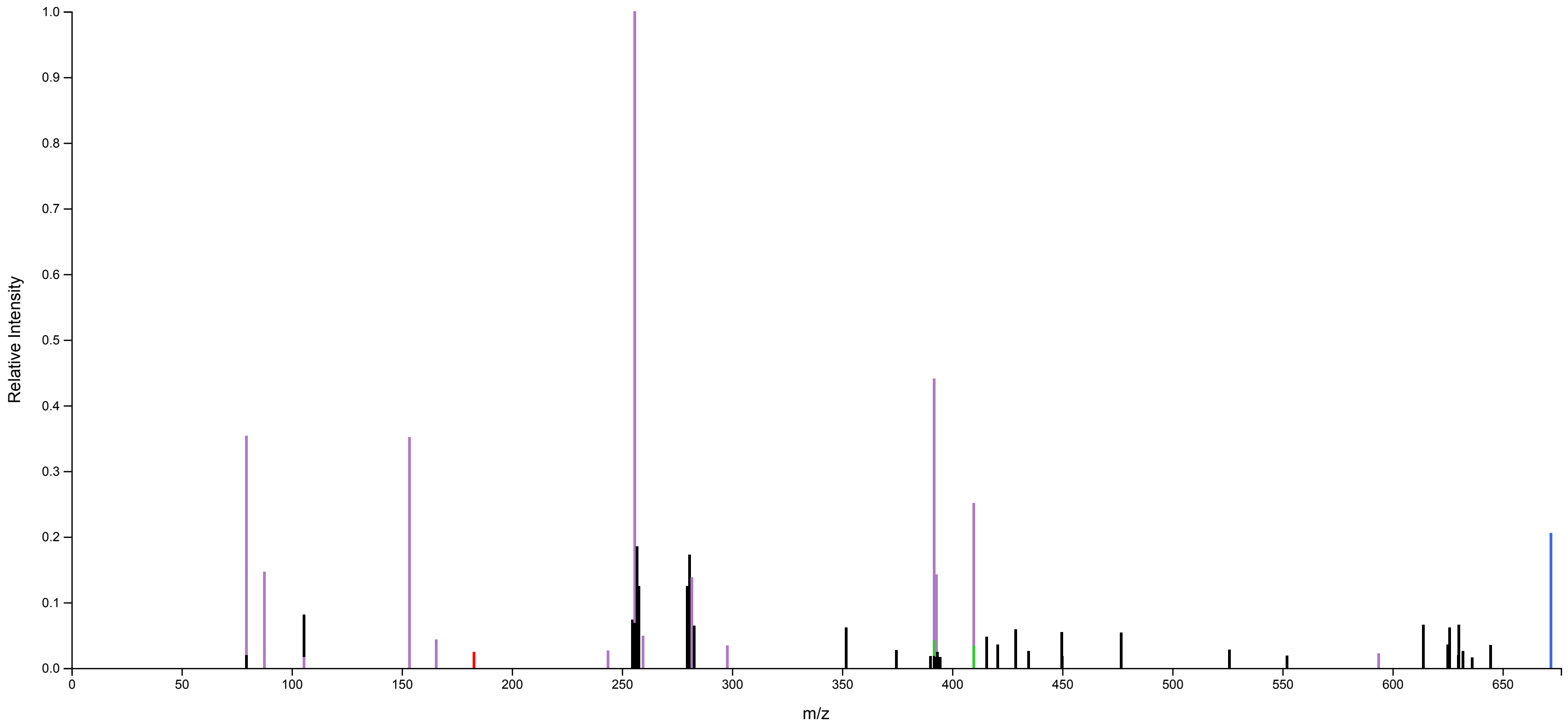

Figure S8. Tandem mass spectrometry (MS/MS) spectral prediction for the feature annotated as phosphatidic acid [PA(16:0/18:2); [M–H]$^{-}$, *m/z* 671.4656, $C_{37}H_{69}O_8P$]. The black trace represents the experimental MS/MS spectrum acquired in the negative ionization mode, whereas the purple trace corresponds to the *in silico* fragmentation prediction generated by SIRIUS. The predicted structure was assigned with 99.58% confidence (CSI:FingerID score: −13,226; XLogP: 12.40), and it is indexed in multiple biochemical databases, including COCONUT, HMDB, and PubChem.

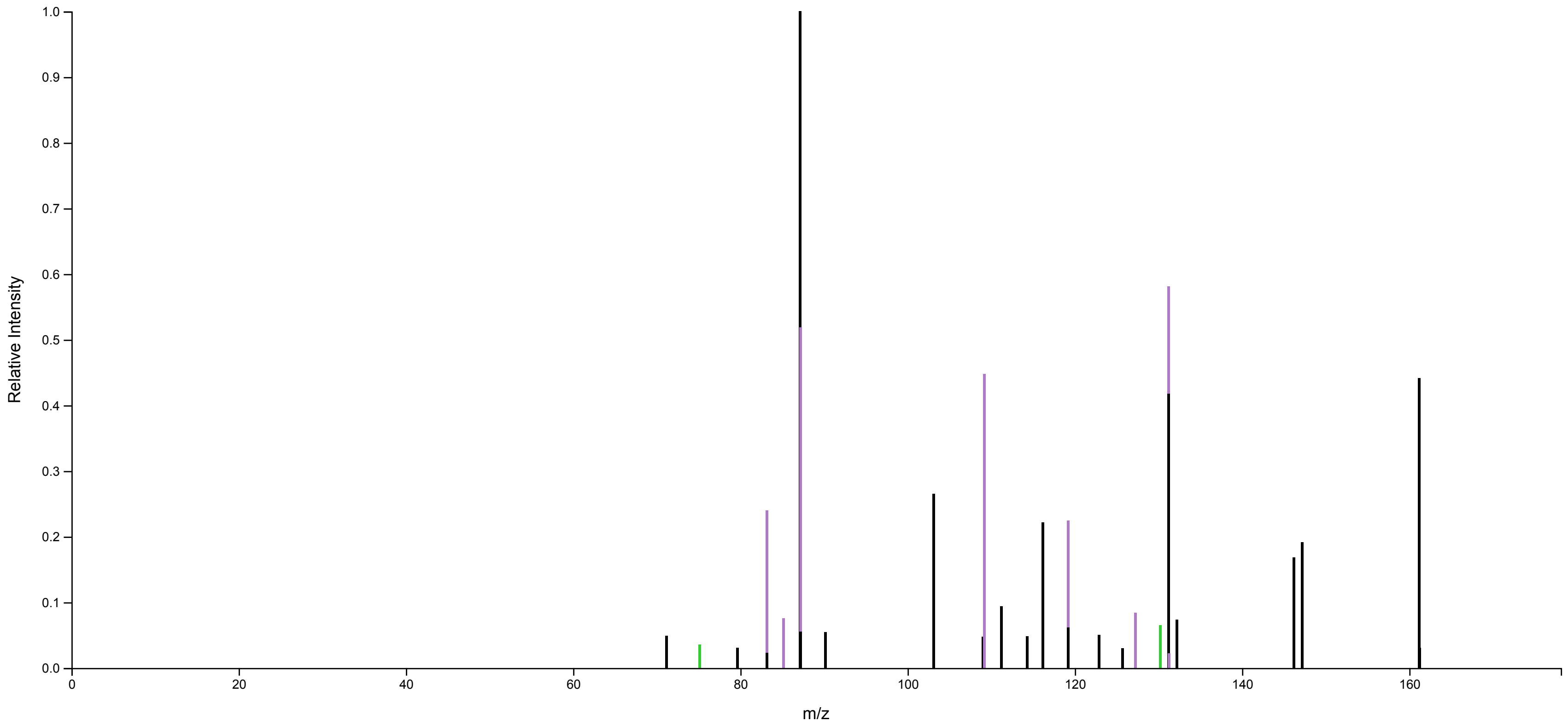

Figure S9. Tandem mass spectrometry (MS/MS) spectral prediction for the feature annotated as 9-hydroxynonanoic acid ($[M–H]^-$, *m/z* 173.1187, $C_9H_{18}O_3$). The black trace represents the experimental MS/MS spectrum acquired in the negative ionization mode, whereas the purple trace corresponds to the *in silico* fragmentation prediction generated by SIRIUS. The predicted structure was assigned with 50.00% confidence (CSI:FingerID score: −66,865; XLogP: 2.07), and it is indexed in natural product and biochemical databases, including COCONUT, PubChem, and KEGG.
